# Synthetic reversed sequences reveal default genomic states

**DOI:** 10.1038/s41586-024-07128-2

**Published:** 2024-03-06

**Authors:** Brendan R. Camellato, Ran Brosh, Hannah J. Ashe, Matthew T. Maurano, Jef D. Boeke

**Affiliations:** 1 Institute for Systems Genetics, NYU Langone Health, New York, NY USA; 2 Department of Pathology, NYU Langone Health, New York, NY USA; 3 Department of Biochemistry and Molecular Pharmacology, NYU Langone Health, New York, NY USA; 4 Department of Biomedical Engineering, NYU Tandon School of Engineering, New York, NY USA

**Keywords:** Transcription, Chromatin, Genomic engineering, Synthetic biology, Genome evolution

## Abstract

Pervasive transcriptional activity is observed across diverse species. The genomes of extant organisms have undergone billions of years of evolution, making it unclear whether these genomic activities represent effects of selection or ‘noise’^1–4^. Characterizing default genome states could help understand whether pervasive transcriptional activity has biological meaning. Here we addressed this question by introducing a synthetic 101-kb locus into the genomes of *Saccharomyces cerevisiae* and *Mus musculus* and characterizing genomic activity. The locus was designed by reversing but not complementing human *HPRT1*, including its flanking regions, thus retaining basic features of the natural sequence but ablating evolved coding or regulatory information. We observed widespread activity of both reversed and native *HPRT1* loci in yeast, despite the lack of evolved yeast promoters. By contrast, the reversed locus displayed no activity at all in mouse embryonic stem cells, and instead exhibited repressive chromatin signatures. The repressive signature was alleviated in a locus variant lacking CpG dinucleotides; nevertheless, this variant was also transcriptionally inactive. These results show that synthetic genomic sequences that lack coding information are active in yeast, but inactive in mouse embryonic stem cells, consistent with a major difference in ‘default genomic states’ between these two divergent eukaryotic cell types, with implications for understanding pervasive transcription, horizontal transfer of genetic information and the birth of new genes.

## Abstract (web summary)

Introduction of a long synthetic DNA into yeast genomic loci results in high default transcriptional activity in yeast but low activity in mouse, suggesting distinct default levels of genomic activity in these organisms.

## Main

The majority of the human genome may be transcribed^1–4^, even though only a small fraction is annotated as discrete mature RNA species^5,6^. Debate remains over whether the approximately 75% of the genome that is covered by detectable transcripts^4^, and the approximately 80% of such transcripts for which there is predicted biochemical activity^2^, represent truly functional activity or random and pervasive ‘noise’^7–9^. In another eukaryotic species, the yeast *S. cerevisiae*, a similar fraction of the genome is transcribed^10^, although the genome is relatively gene-dense with an average intergenic distance^11^ of around 400 bp compared with the approximately 100,000 bp in the human genome^12^. This raises the question of whether all eukaryotic genomes are transcribed at the same level, regardless of their structure. Understanding the ‘default state’ of a genome—that is, the way a sequence lacking evolved features is acted on by the host—would be useful in interpreting the meaning of such transcriptional activity.

A genome that is active by default would present ample opportunity for transcriptional machinery to bind non-specifically, leading to spurious activity, whereas a genome that is inactive by default would generally preclude such low-specificity activity. The true default state of a genome, if such a thing exists, is difficult to determine, owing to billions of years of evolutionary pressure that has acted on existing sequences. It is thus unclear to what extent observed genomic states are passively present by default, or actively produced by chromatin-interacting proteins that recognize specific sequences selected for over time. A true default genomic state can be queried by observing activity of a newly introduced, evolutionarily naive locus. Indeed, a hypothetical ‘random genome’ experiment has been proposed as the ideal negative control for interpreting reports of large-scale genomic activity^13^, in which megabase-sized fragments of random DNA can be introduced into a cell and its activity compared with that of the endogenous genome. However, owing to technical limitations, such experiments have not yet been performed.

To date there has not been any well-controlled characterization of novel DNA loci in mammalian genomes, or a comparison of genomic activity for the same locus in different organismal contexts. Current techniques in synthetic genomics enable the design, assembly and delivery of very large pieces of DNA^14,15^. Locus-scale DNA constructs, up to hundreds of kilobases long, can be assembled de novo in yeast assembly vectors (YAVs), which exist as episomal DNA circles separate from native yeast and bacterial genomes. The ability to synthesize large DNA loci de novo enables complete design freedom over the sequence of synthetic DNA, although this realization has been limited in practice. In recent years, we have developed a workflow for synthetic regulatory genomics involving the de novo assembly of large DNA loci, including an intermediate step involving *S. cerevisiae*, for delivery and characterization in a desired eukaryotic context, typically mouse embryonic stem (ES) cells^16–20^. This enables straightforward design and assembly of novel DNA loci that do not exist in nature, and characterization of such loci in the distinct genomic contexts of *S. cerevisiae* and *M. musculus*. By introducing novel DNA loci to both yeast and mouse cells we can compare the default genomic states of two distinct eukaryotic hosts. Here we decided on a simple yet informative approach: to write an entire locus backwards as an initial foray into exploration of the behaviour of truly random sequences in distinct types of living cells, and an initial approximation of a ‘random genome’ experiment.

## Engineering of synthetic loci

**Fig. 1:**
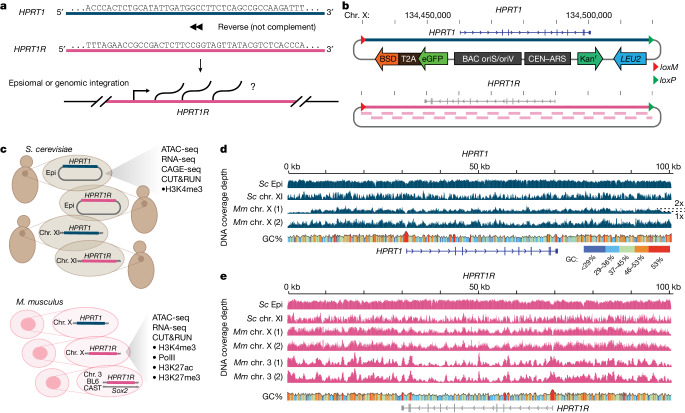
Design and construction of synthetic *HPRT1* and *HPRT1R*. **a**, Schematic illustrating the strategy of reversing the *HPRT1* sequence to produce the *HPRT1R* sequence. **b**, The human *HPRT1* locus was cloned into a assemblon vector and flanked by* lox* recombination sites for Big-IN integration. *HPRT1R* was assembled de novo from 28 synthetic segments, shown below the locus. Vector components include centromere (CEN)–autonomously replicating sequence (ARS) and *LEU2* for propagation and selection in *S. cerevisiae*, bacterial artificial chromosome (BAC) oriS and oriV (low copy and inducible high copy origins, respectively) and the kanamycin resistance gene (Kan^r^) for propagation and selection in *Escherichia coli*, and eGFP–T2A–BSD for transient selection in mammalian cells. Chr., chromosome. **c**, Genomic contexts for interrogating synthetic locus activity. Episomal (Epi) and genomically integrated (chromosome XI) in *S. cerevisiae*, and genomically integrated (chromosome X and chromosome 3) in *M. musculus*. The chromosome 3 integration is monoallelic on the BL6 locus, leaving *Sox2* intact on the CAST locus. **d**,**e**, DNA sequencing coverage plots from next-generation sequencing verification of assembled and integrated synthetic loci. Yeast samples were whole-genome sequenced and mouse ES cell samples were characterized by Capture-seq. *Sc* Epi, episomal in *S. cerevisiae*; *Sc* chr. XI, integrated on *S. cerevisiae* chromosome XI; *Mm* chr. X, integrated on *M. musculus* chromosome X; *Mm* chr. 3, integrated on *M. musculus* chromosome 3. (1) and (2) indicate two independent mouse ES cell clones. GC content shown as a line plot and colour-scaled. For *HPRT1 Mm* chr. X (1), dotted lines on the right show 2x coverage depth for most of the synthetic locus and 1x coverage depth at the edges. The relative position of the reversed *HPRT1* coding sequence is indicated above the BAC in **b** and below the coverage plots in **e**.

To design a novel large piece of DNA, we reversed the sequence of the human hypoxanthine phosphoribosyltransferase 1 (*HPRT1*) locus (Fig. 1a). By using the reverse sequence (not the reverse complement), which we refer to as *HPRT1R*, we ensured that the new locus lacks coding information but retains sequence features such as GC content, homopolymer runs and repeat frequency and position, that might otherwise confound analysis. This approach also provides a forward, coding ‘control’ locus, the natural *HPRT1* sequence, which we previously synthesized and delivered to mouse ES cells, where it was expressed^18^. Statistics describing sequence composition of both synthetic loci (Table 1) indicate that although reversing the *HPRT1* sequence ablates evolved regulatory elements, many potentially functional sequences, which have low information content, are still present and might be expected to occur by chance in DNA sequences of sufficient length.

**Table 1:** Sequence featured of the synthetic locus

		Synthetic locus		
	Feature	*HPRT1*	*HPRT1R*	*HPRT1R*^*noCpG*^
	Length	100,667	100,667	95,067
	GC content	0.41	0.41	0.38
**Dinucleotides**	AA	6,601	6,601	6,601
	AC	4,942	6,952	5,803
	AG	7,012	5,734	6,883
	AT	7,491	6,759	6,759
	CA	6,952	4,942	5,743
	CC	4,261	4,261	3,442
	CG	1,202	4,499	0
	CT	7,038	5,751	6,583
	GA	5,734	7,012	6,211
	GC	4,499	1,202	726
	GG	4,348	4,348	4,455
	GT	5,368	7,388	6,556
	TA	6,759	7,491	7,491
	TC	5,751	7,038	5,797
	TG	7,388	5,368	6,609
	TT	7,415	7,415	7,415
**CpG**	Expected	4,291	4,291	3,386
	Ratio	0.28	1.05	0
	Yeast TFBSs^a^	5,159	18,191	13,284
Mouse TFBSs^b^	p-val < 0.001	24,578	19,782	21,897
	q-val < 0.01	1,132	707	882

^a^Yeast TFBSs as predicted using the YEASTRACT+ database^65^.

^b^Mouse TFBSs as predicted using FIMO^66^ in the MEME suite with the JASPAR vertebrate motif database^67^.

**Extended Data Fig. 1:**
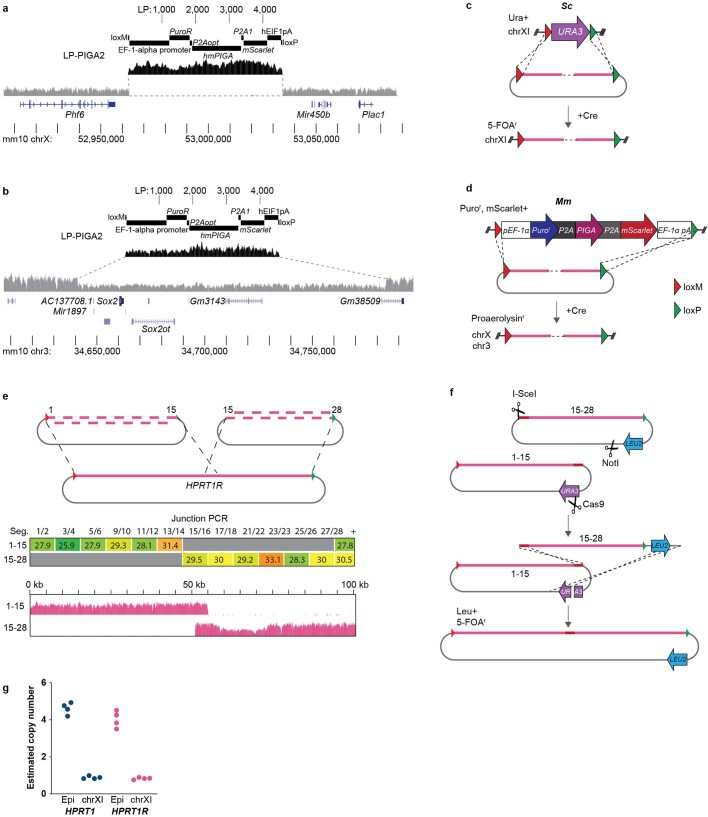
Synthetic locus assembly and cell line engineering. **a,b**, Landing pad installed on the X chromosome (**a**) or chromosome 3 (**b**) of the mouse genome. Presence of the landing pad was verified by Capture-seq (black track) on DNA from mESCs, as was the deletion of the endogenous *Hprt* locus, or one *Sox2* locus (grey track), prior to synthetic locus delivery. Components of the landing pad are indicated above the sequencing track. **c**, Strategy for integrating synthetic loci into the yeast (*Sc*) genome. A landing pad containing the *URA3* selectable/counterselectable marker cassette and flanked by heterotypic lox sites was integrated at YKL162C-A on chrXI by integrative homologous recombination in strains already harboring an episomal *HPRT1* or *HPRT1R* assemblon. The synthetic loci were then recombined into this locus using transient Cre expression, overwriting the *URA3* landing pad. **d**, Strategy for integrating synthetic loci into the mouse (*Mm*) genome. A landing pad containing a selectable/counterselectable cassette flanked by heterotypic lox sites was installed on chrX or chr3, overwriting the endogenous *Hprt* or one *Sox2* allele, respectively. The synthetic loci were then delivered to these loci using Cre-mediated cassette exchange, overwriting the landing pad. **e**, Assembly of *HPRT1R* in two parts, the first containing segments 1-15 and the second segments 15-28. An example junction-qPCR verification is shown, reporting Ct values for qPCR reactions using primers for the indicated junctions (*i.e*., junction 1/2 is the junction between segments 1 and 2) performed on whole yeast DNA following assembly transformation and selection. The last column represents a positive control reaction detecting a yeast genomic marker. Sequencing coverage plots for the two half-assemblies from yeast whole genome sequencing is shown below. **f**, The eSwAP-In^18^ (extrachromosomal Switching Auxotrophies for Progressive Integration) strategy for combining the two *HPRT1R* half-assemblies. Segments 15-28 and the *LEU2* marker are combined with segments 1-15 and the rest of the vector backbone through homologous recombination using the common segment 15 and common sequence downstream of the selection markers as homology arms to promote recombination. Positive recombinants are selected as Leu+ and 5-FOA^r^ (Ura–). **g**, Estimated copy number of *HPRT1* and *HPRT1R* in yeast when present episomally (Epi) or integrated (chrXI). Each data point represents the ratio of average whole genome sequencing coverage depth over the synthetic locus divided by the average coverage depth over the yeast genome for independently-sequenced yeast clones.

The synthetic *HPRT1* and *HPRT1R* loci, hereafter referred to as assemblons, were assembled in yeast assembly vectors (Fig. 1b) (see Supplementary Table 1 for details of the YAVs). YAVs facilitate Cre-mediated delivery into landing pads pre-installed in the yeast or mouse genomes (Extended Data Fig. 1a–d) (see Supplementary Table 2 for details of the landing pad), enabling readout in four contexts: in yeast, as episomes and genomic integrants, and in mouse ES cells, as genomic integrants at two distinct genomic locations (Fig. 1c). The *HPRT1* locus was shuttled from a previous assemblon^18^ into a YAV allowing Big-IN delivery^17^, and the synthetic *HPRT1R* locus was assembled de novo from synthetic DNA pieces (Extended Data Fig. 1e,f) (see Supplementary Table 3 for synthetic DNA sequences, and Supplementary Table 4 for oligonucleotide sequences). All assemblons were verified by next-generation sequencing (Fig. 1d,e). The synthetic loci were integrated into the yeast *YKL162C-A* gene, a previously identified safe harbour site^21^, and into the mouse genome by overwriting the *Hprt1* locus on the X chromosome, and at *Sox2*, overwriting one endogenous allele on chromosome 3 (Extended Data Fig. 1a,b) (specific genomic coordinates in Supplementary Table 5). Successful integrants were isolated and ultimately verified by whole-genome sequencing in yeast and targeted resequencing^17^ (Capture-seq) in mouse ES cells (Fig. 1d,e). The Capture-seq protocol involves targeted sequencing of genomic regions flanking the integration site, enabling copy number estimation of integrated loci based on comparison to the flanking regions, which have a single copy of the *Hprt1* site on the X chromosome (the BL6xCAST mouse ES cells used are male with an XY karyotype) and two copies of the *Sox2* site on chromosome 3. Analysis of Capture-seq data showed that one mouse ES cell clone had synthetic *HPRT1* integrated as two copies (Fig. 1d), whereas all other synthetic loci were integrated as single copies. mouse ES cells with successful integration of synthetic *HPRT1* were also selected for their ability to grow in hypoxanthine-aminopterin-thymidine (HAT)-supplemented medium^22^, demonstrating that *HPRT1*, which was previously shown to be expressed in mouse cells^18^, is able to functionally complement the loss of mouse *Hprt1*.

## Synthetic loci are active in yeast

**Fig. 2:**
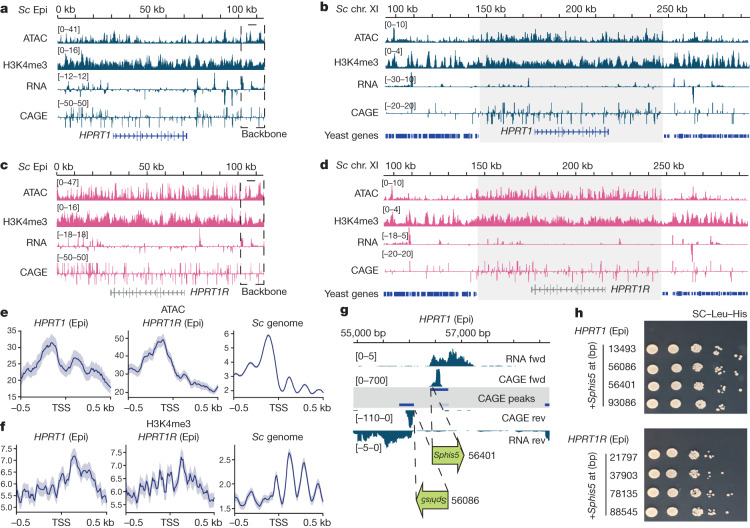
Synthetic *HPRT1* and *HPRT1R* loci are active in yeast. **a**,**b**, Sequencing tracks for ATAC-seq, H3K4me3 CUT&RUN, RNA-seq and CAGE-seq reads aligned to the synthetic *HPRT1* locus as an episome (Epi) (**a**) or integrated on chromosome XI (**b**). **c**,**d**, Sequencing tracks of ATAC-seq, H3K4me3 CUT&RUN, RNA-seq and CAGE-seq reads aligned to the synthetic *HPRT1R* locus as an episome (**c**) or integrated on chromosome XI (**d**). The synthetic locus regions (*HPRT1* and *HPRT1R*) are shaded in **b**,**d**. The *HPRT1* coding sequence is indicated in **a**,**b** and the relative position corresponding to the reversed coding sequence is indicated in **c**,**d**. For loci integrated into chromosome XI (**b**,**d**), approximately 50 kb of flanking yeast genome is shown upstream and downstream of the integrated synthetic loci with annotated yeast genes indicated. RNA-seq and CAGE-seq tracks are stranded, displayed with reverse strand reads inverted and below forward strand reads. Sequencing tracks are shown for one replicate for each genomic context. **e**,**f**, Metaplots of ATAC-seq (**e**) and H3K4me3 CUT&RUN (**f**) signal at the TSS (defined by experimental CAGE-seq peaks) ±0.5 kb for *HPRT1* and *HPRT1R* episomal assemblons as well as the yeast (*Sc*) genome. Shaded region shows standard error. **g**, Example strategy for insertion of the *Sphis5* coding sequence at two experimentally identified TSSs. RNA-seq and CAGE-seq tracks are shown, as well as CAGE-seq peaks. The *Sphis5* coding sequence (green arrow) is inserted with the 5′ untranslated region at the 5′ boundary of the CAGE-seq peak. Fwd, forward; rev, reverse. **h**, Spot assays for yeast with *Sphis5* integration on the *HPRT1* or *HPRT1R* episome, and their parental strains, on SC–Leu–His medium. For *Sphis5* insertion strains, the number indicates the position of the *Sphis5* insertion along the synthetic locus.

**Extended Data Fig. 2:**
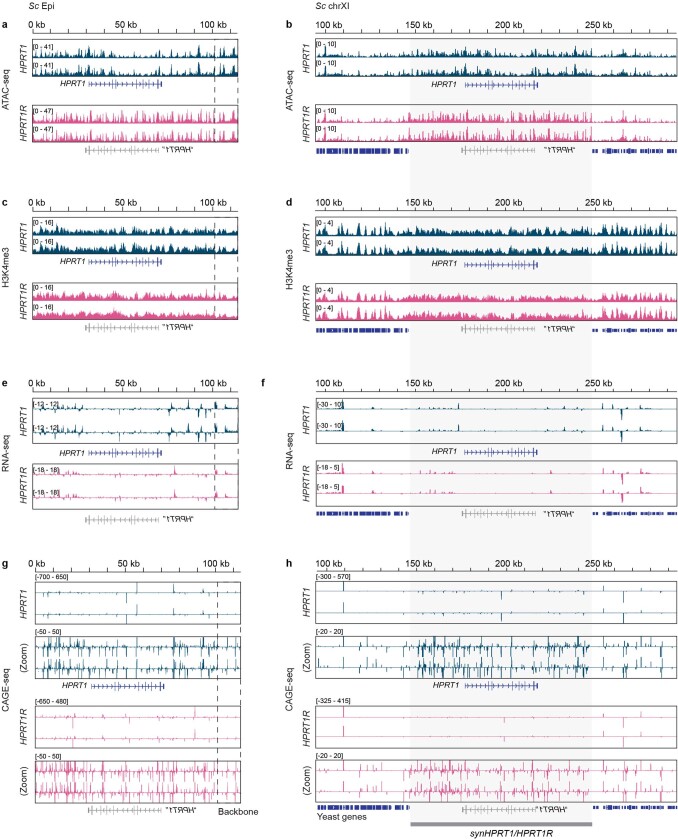
Yeast sequencing assay replicate tracks. Sequencing tracks for ATAC-seq (**a,b**), H3K4me3 CUT&RUN (**c,d**), RNA-seq (**e,f**), and CAGE-seq (**g,h**) at *HPRT1* and *HPRT1R* in yeast. Tracks are shown for episomal (Epi, **a,c,e,g**) and chromosomally integrated (chrXI, **b,d,f,h**) synthetic loci in two biological replicates for each strain. The *HPRT1* coding sequence is indicated for the *HPRT1* locus, and the relative position corresponding to the reversed coding sequence is indicated for the *HPRT1R* locus. The synthetic locus region is shaded for chromosomally integrated loci, and ~50 kb of flanking yeast genome is included, with annotated yeast genes indicated. RNA-seq (**e,f**) and CAGE-seq (**g**, h) tracks are stranded, displayed with reverse strand reads inverted and below forward strand reads. For CAGE-seq (**g,h**), “(Zoom)” tracks show the same data as the tracks above for each strain with a smaller y-axis scale to better visualize smaller peaks.

**Extended Data Fig. 3:**
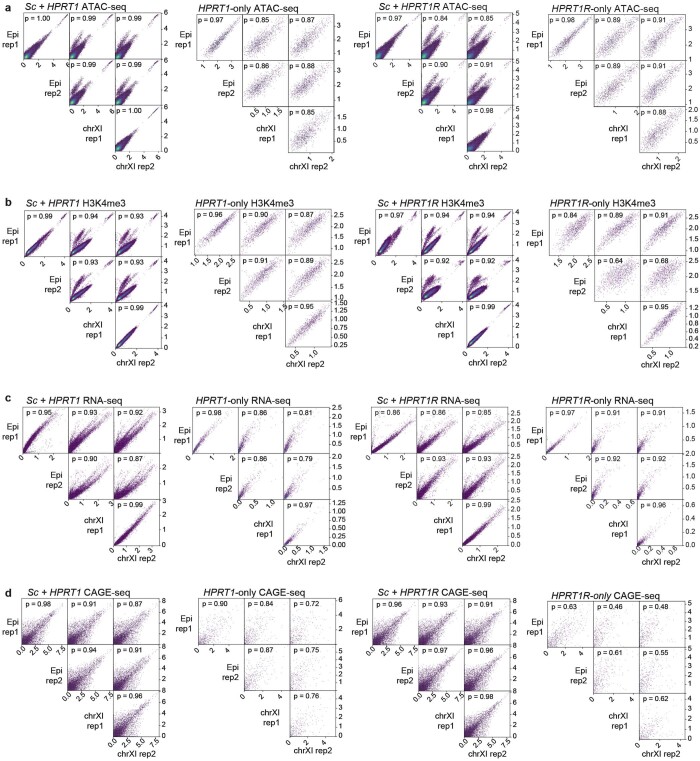
Yeast sequencing assay replicate correlates. Scatterplots showing correlation between ATAC-seq (**a**), H3K4me3 CUT&RUN (**b**), RNA-seq (**c**), and CAGE-seq (**d**) signal (bigWig scores) over 100 bp windows for the combined synthetic locus and yeast genome (*Sc* + *HPRT1/HPRT1R*) and for the synthetic locus only (*HPRT1*/*HPRT1R*-only). Both replicates for each of the episomal (Epi) and chromosomally integrated (chrXI) synthetic loci are compared, and Pearson’s correlation (p) between each replicate is reported.

**Extended Data Fig. 4:**
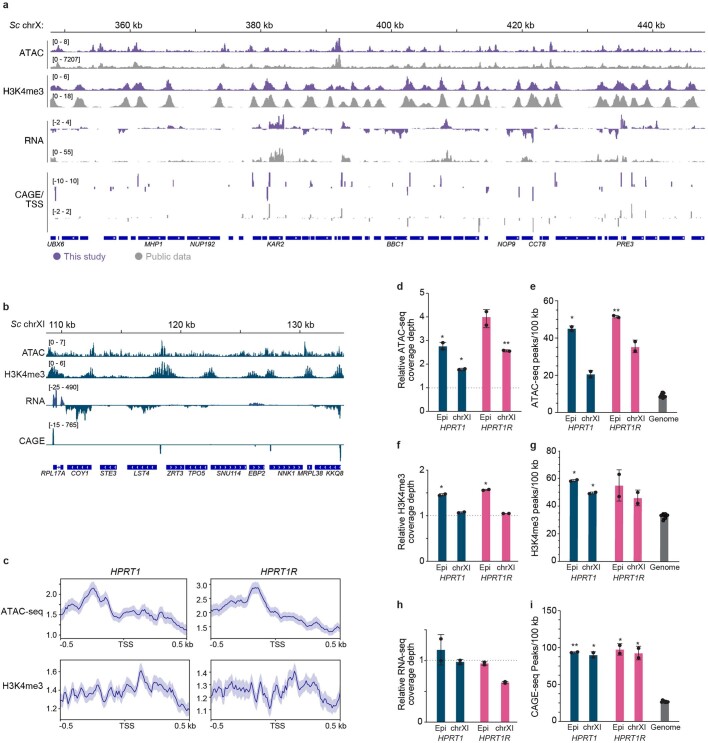
Yeast sequencing assays supplement. **a**, Snapshot of a region on *S. cerevisiae* chromosome X showing sequencing data for ATAC-seq, H3K4me3 CUT&RUN, RNA-seq, and CAGE-seq from this study (purple) and data for ATAC-seq, H3K4me3 ChIP-seq, RNA-seq, and CAGE-seq from publicly available, published datasets (grey). Data from this study is from one replicate yeast strain with *HPRT1* integrated on chrXI. GEO accession numbers for the public data: ATAC-seq GSM6139041, H3K4me3 ChIP-seq GSM3193266, RNA-seq GSM5702033, yeast CAGE-seq^96^. **b**, Sequencing tracks for ATAC-seq, H3K4me3 CUT&RUN, RNA-seq, and CAGE-seq for the region in the yeast genome just upstream of the *HPRT1*/*HPRT1R* integration site. Annotated yeast genes are shown below. Data is from one replicate yeast strain with *HPRT1* integrated on chrXI. **c**, Metaplots of ATAC-seq and H3K4me3 CUT&RUN signal at the transcription start site (TSS, defined by experimental CAGE-seq peaks) +/− 0.5 kb for synthetic *HPRT1* and *HPRT1R* loci integrated into the yeast genome on chrXI. Shaded region shows standard error. **d,f,h**, Relative coverage depth for ATAC-seq (**d**), H3K4me3 CUT&RUN (**f**), and RNA-seq (**h**) of the synthetic loci as episomes (Epi) and chromosomally integrated (chrXI) compared to the genome average. For episomes, coverage was corrected for the estimated copy number of the episomes. **e,g,i**, Peak counts per 100 kb for ATAC-seq (**e**), H3K4me3 CUT&RUN (**g**), and CAGE-seq (**i**) for the synthetic loci as episomes (Epi) and chromosomally integrated (chrXI) as well as the genome average. Bars show mean of biological replicates +/- SD. * p < 0.05, ** p < 0.01, two-tailed paired t-test comparing *HPRT1*/*HPRT1R* to the yeast genome average.

We first assessed activity of the novel synthetic loci in yeast, both as episomes and as chromosomal integrations (yeast strain details in Supplementary Table 6). For sequencing-based assays, replicates agreed well, as assessed by Pearson correlation of genome-wide signal depth (Extended Data Figs. 2 and 3) and by comparison with publicly available sequencing data for the same or similar assays (Extended Data Fig. 4a). Assaying chromatin accessibility by assay for transposase-accessible chromatin using sequencing (ATAC-seq), we observed multiple peaks of highly accessible chromatin across the entire synthetic locus for both *HPRT1* and *HPRT1R* (Fig. 2a–d and Extended Data Fig. 2a,b). Using an adjacent region of the yeast genome as a reference, ATAC-seq peaks coincided with promoter regions of transcribed genes (Extended Data Fig. 4a,b). For both *HPRT1* and *HPRT1R* synthetic loci, the highly accessible regions were conserved across replicates and between episomal and integrated loci (Extended Data Fig. 2a,b). Average ATAC-seq coverage depth was greater across the synthetic *HPRT1* and *HPRT1R* loci compared with the genome average calculated over 100-kb sliding windows (Extended Data Fig. 4d), which was also evident when comparing the integrated synthetic loci to their surrounding native genomic regions (Fig. 2b,d and Extended Data Fig. 2b). ATAC-seq coverage depth was also greater for episomal loci compared with integrated loci, even when normalizing for estimated copy number based on DNA sequencing coverage (Extended Data Figs. 1g and 4d). The synthetic loci contained more ATAC-seq peaks over 100 kb compared with the genome average (Extended Data Fig. 4e), and although we observed peaks coinciding with the *HPRT1* transcription start site (TSS), and the relative TSS position in *HPRT1R*, peaks generally did not correspond with known *HPRT1* functional elements.

We next checked for H3K4me3 at the synthetic loci, a marker of active transcription of nearby genes. Using cleavage under targets & release using nuclease^23^ (CUT&RUN), we found broad coverage of H3K4me3 over both synthetic loci (Fig. 2a–d and Extended Data Fig. 2c,d). H3K4me3 coverage of *HPRT1* and *HPRT1R* appears broader than coverage over the yeast genome, where tight peaks coincide with known promoter regions (Extended Data Fig. 4a,b). H3K4me3 coverage was also greater across episomal *HPRT1* and *HPRT1R* loci compared to the yeast genome average and the synthetic loci contained more H3K4me3 peaks per 100 kb than the genome average (Extended Data Fig. 4f,g).

**Extended Data Fig. 5:**
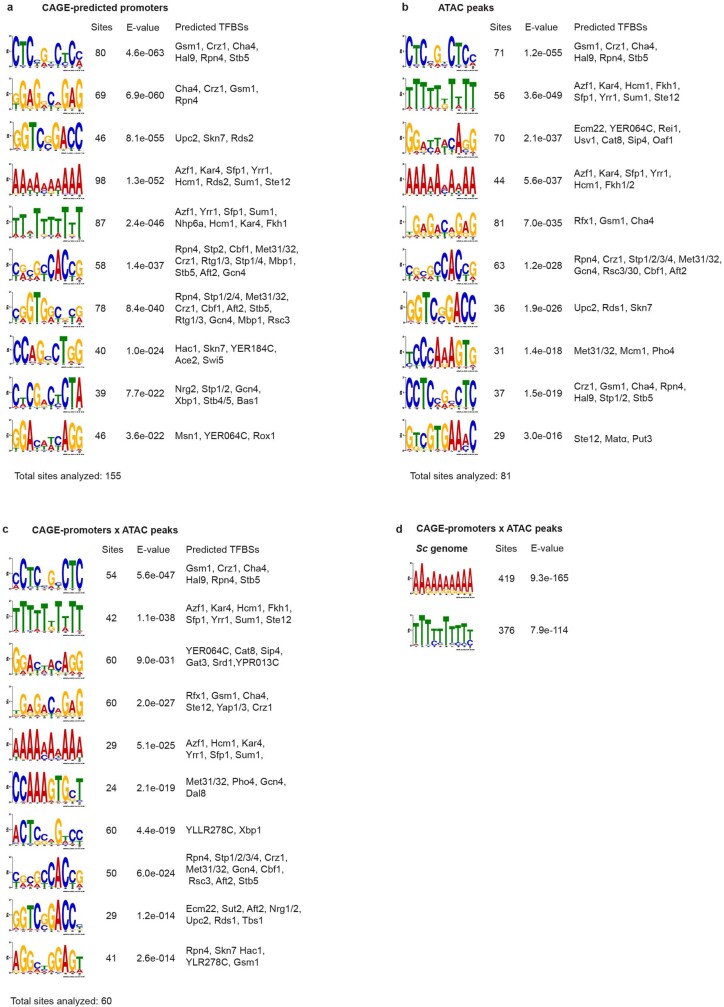
Sequencing motifs analysis for HPRT1 and HPRT1R in yeast. **a-c**, The top 10 motifs identified across both synthetic *HPRT1* and *HPRT1R* loci, within predicted promoter regions, defined as 200 bp upstream and 100 bp downstream of the experimentally identified CAGE-seq peaks (**a**), ATAC-seq peaks (**b**), and ATAC-seq peaks that overlap with predicted promoters (**c**). **d**, The only two motifs identified genome-wide in ATAC-seq peaks overlapping with CAGE-predicted promoters. Motifs were identified using MEME with a max width of 10 bp. For each set of motifs, the number of identified sites is reported, as well as the total number of sites analyzed, the E-value, and predicted transcription factor binding sites (TFBSs), identified using Tomtom in the MEME suite.

To determine whether observed chromatin accessibility and H3K4me3 patterns relate to transcription, we performed RNA sequencing (RNA-seq) for strains with episomal and chromosomally integrated synthetic loci (Fig. 2a–d and Extended Data Fig. 2e,f). RNA-seq reads mapped across both *HPRT1* and *HPRT1R* synthetic loci, and RNA-seq peaks were consistent between replicates and between episomal and integrated loci. The synthetic loci showed RNA-seq coverage depth similar to the genome average, which is gene-dense (Extended Data Fig. 4h). We used cap analysis gene expression and sequencing^24^ (CAGE-seq) to map TSSs (Fig. 2a–d and Extended Data Fig. 2g,h), and found that both synthetic loci have around 3 times more CAGE-seq peaks per 100 kb than the yeast genome average (Extended Data Fig. 4i). CAGE-seq peaks map to the 5′ end of annotated and expressed yeast genes (Extended Data Fig. 4a,b), adding confidence that peaks observed in the synthetic loci are true TSSs. Using the 5′ boundary of CAGE-seq peaks as reference points, we produced metaplots of ATAC-seq and H3K4me3 signals for TSSs in the synthetic *HPRT1* and *HPRT1R* loci and throughout the yeast genome (Fig. 2e,f and Extended Data Fig. 4c). The metaplot profiles for the synthetic loci generally match that for the yeast genome and, although they lack the precise nucleosome repeat definition seen in genome profiles obtained by averaging tens of thousands of TSSs, rough periodicity can be observed. RNA-seq and CAGE-seq signals do not appear to correspond to known gene features in the *HPRT1* locus, and in fact appear to be generally depleted around the *HPRT1* transcription unit and its corresponding region in the *HPRT1R* locus. We performed motif analysis using MEME^25^ on the predicted promoter regions—defined as 200 bp upstream and 100 bp downstream of identified TSSs—on ATAC-seq peaks and on ATAC-seq peaks that overlap predicted promoters (Extended Data Fig. 5). We identified a number of significantly enriched sequence motifs, including stretches of A and T reported to precede TSSs^26^, as well as the dyad symmetric CTCNGNCTC/GAGNCNGAG motif, and the palindromic GGTC(G/C)GACC/CCAG(C/G)CTGG motif. Although the identified motifs do not exactly match known transcription factor binding sites (TFBSs), predicting TFBSs using Tomtom^27^ identified a number of potential sites for stress-responsive transcription factor including Crz1, Rpn4 and Gsm1. Performing the same analysis on overlapping CAGE-predicted promoters and ATAC-seq peaks genome-wide, the only significant motifs identified are poly-A and poly-T regions.

**Extended Data Fig. 6:**
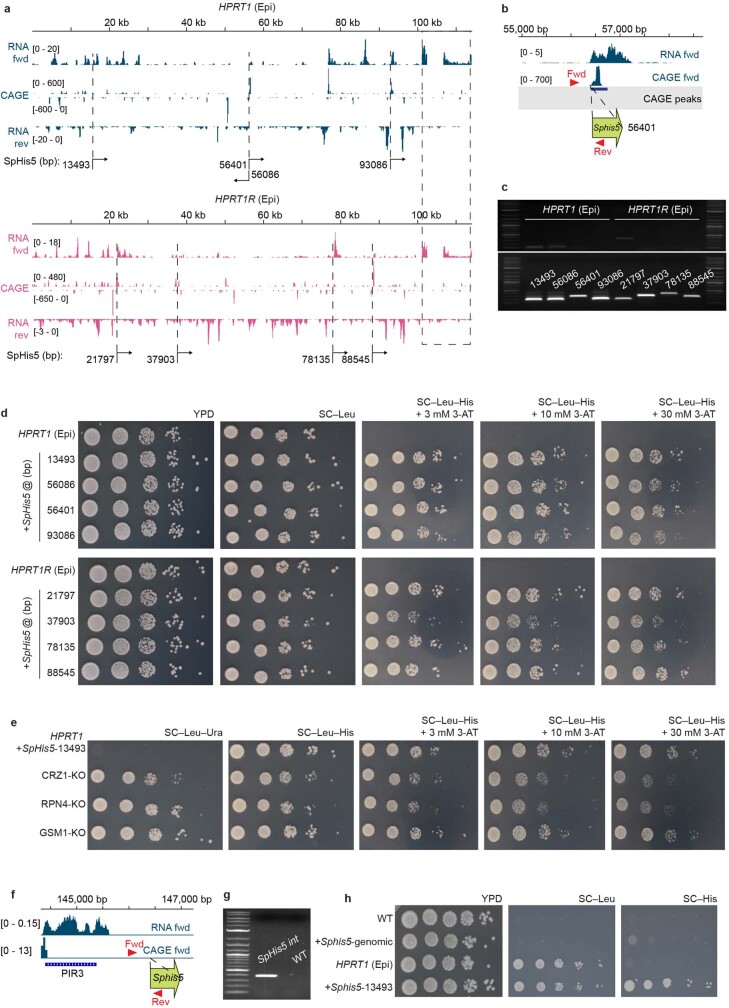
Insertion and characterization of Sphis5 at transcription start sites in synthetic HPRT1 and HPRT1R loci. **a**, Identification of *Sphis5* insertion sites based on experimental RNA-seq and CAGE-seq. Sequencing tracks for *HPRT1* (top, blue) and *HPRT1R* (bottom, pink) are shown with forward reads on the top and reverse reads underneath and inverted. *Sphis5* insertion sites are indicated with black dashed lines across the sequencing tracks. The precise insertion position, in base-pairs, and direction of transcription is indicated below the sequencing tracks. **b**, Example strategy for insertion of the *Sphis5* coding sequence at a third experimentally-identified transcription start site. RNA-seq and CAGE-seq sequencing tracks are shown, as well as CAGE-seq peaks. The *Sphis5* coding sequence (green arrow) is inserted with the 5’UTR at the 5’ boundary of the CAGE-seq peak. **c**, PCR verification of on-target *Sphis5* insertion. PCRs were performed using a forward primer outside of the *Sphis5* coding sequence and a reverse primer inside the *Sphis5* coding sequence (as indicated by red arrows in **b**) for each insertion position. PCRs were performed on the parental yeast strain with just the *HPRT1* or *HPRT1R* episome (Epi, top panel) and on the derivative clones with *Sphis5* inserted at the indicated position, in base-pairs (bottom panel). **d**, Spot assays of parental *HPRT1* and *HPRT1R* episome-containing yeast strains, and their derivative *Sphis5* insertion strains, with the position of the *Sphis5* insertion is indicated in base-pairs. Yeast were spotted on YPD, SC–Leu, and SC–Leu–His with 3-amino-1,2,4-triazole (3-AT), a competitive inhibitor of the *Sphis5* gene product, added at the indicated concentrations. **e**, Spot assays of the *HPRT1* strain with *Sphis5* inserted at 13493 bp and derivatives in which transcription factors were knocked out by *URA3* integration. Yeast were spotted on SC–Leu–Ura and SC–Leu–His with 3-AT added at the indicated concentrations. **f**, Insertion of the *Sphis5* coding sequence into the yeast genome at the YKL162C-A locus, not adjacent to a transcription start site, as a negative control. **g**, PCR verification of on-target *Sphis5* insertion into the genome using a primer pair as indicated by red arrows in **f**. **h**, Spot assays of the WT parental yeast strain, that strain with *Sphis5* inserted into the genome, with the *HPRT1* episome, and with *Sphis5* inserted into the *HPRT1* episome at 13493 bp. Yeast were spotted on YPD, SC–Leu, and SC–His. Residual background growth of the strains with *Sphis5* inserted into the genome and in cells with the *HPRT1* episome (lacking *Sphis5*) reflects the fact that yeast cells contain large stores of vacuolar amino acids which allow for a limited number of cell divisions on selective medium; the slightly higher growth in the former presumably reflects exceedingly low levels of *Sphis5* transcription.

To assess whether observed sites of transcription initiation can produce functional mRNAs, we cloned the *his5* transcription unit from *Schizosaccharomyces pombe* (*Sphis5*) downstream of the predicted promoters for eight identified transcription start sites (Fig. 2g and Extended Data Fig. 6a–c). As the parental BY4741 strain is His^*–*^, only yeast expressing the *Sphis5* transgene can survive on histidine dropout medium. We observed His^*+*^ colonies following transformation-mediated integration of the *Sphis5* gene into four sites each of the *HPRT1* and *HPRT1R* episomes and confirmed the His^*+*^ phenotype compared with the parental yeast strains (Fig. 2h and Extended Data Fig. 6d), demonstrating that novel TSSs are able to drive transcription of functional mRNAs and proteins. All eight tested sites appear to produce sufficiently high levels of transcription, as there were no observable fitness differences between the *Sphis5* strains, even when grown in the presence of 3-amino-1,2,4-triazole (3-AT), a competitive inhibitor of the *Sphis5* gene product used to titrate relatively small differences in gene expression. To determine whether any transcription factors predicted to bind to the putative promoter region motifs are responsible for transcription from the integrated transgenes, we deleted three candidate factor genes, *CRZ1*, *RPN4* and *GSM1*, from yeast strains with *Sphis5* inserted at *HPRT1* −13493. This putative promoter region has the CTCNGNCTC/GAGNCNGAG motif that is predicted to bind these non-essential transcription factors. After identifying successful knockouts, we observed specific reduction in growth in the absence of histidine for the CRZ1 and RPN4 knockouts (Extended Data Fig. 8e).

## Synthetic loci are inactive in mouse ES cells

**Fig. 3:**
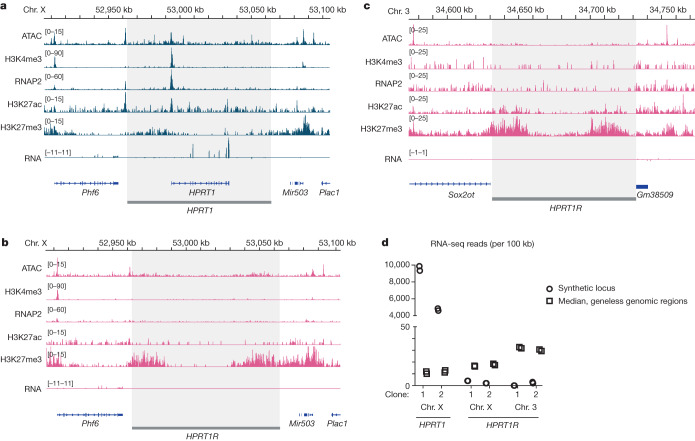
Synthetic *HPRT1* is active, whereas *HPRT1R* is inactive, in mouse ES cells. **a**–**c**, Sequencing tracks for ATAC-seq, H3K4me3, RNAP2, H3K27ac and H3K27me3 CUT&RUN, and RNA-seq over the synthetic *HPRT1* and *HPRT1R* loci integrated into the mouse genome. One clonal replicate is shown for each integration: *HPRT1* chromosome X (2) (**a**), *HPRT1R* chromosome X (1) (**b**) and *HPRT1R* chromosome 3 (1) (**c**). Synthetic locus regions (*HPRT1* and *HPRT1R*) are shaded. The *HPRT1* coding sequence is indicated in **a**. Approximately 50 kb of flanking mouse genome is included, with annotated mouse genes indicated. RNA-seq tracks are stranded, displayed with reverse strand reads inverted and below forward strand reads. **d**, RNA-seq read counts for the synthetic loci (circles) and for 100-kb geneless regions of the mouse genome (squares, median of 70,107 100-kb sliding windows).

**Extended Data Fig. 7:**
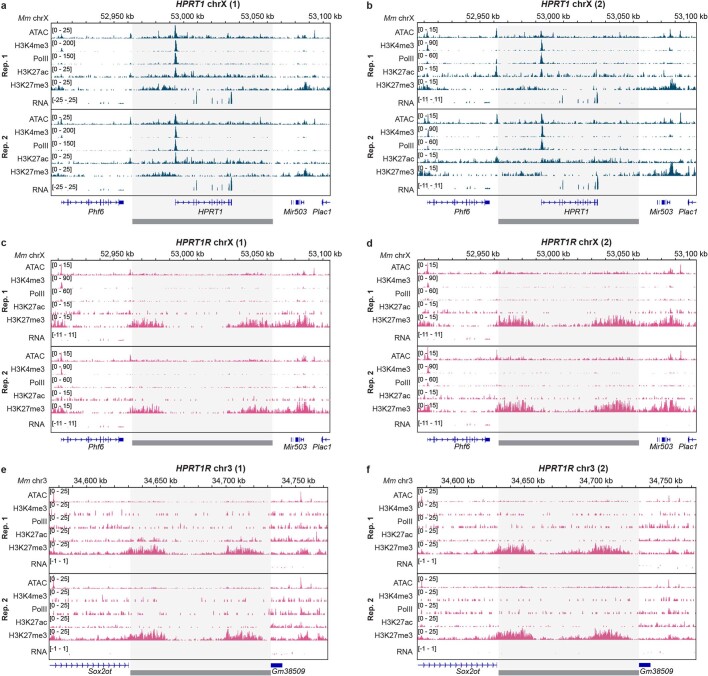
*HPRT1*, *HPRT1R* in mESCs replicate tracks. Sequencing tracks for ATAC-seq, H3K4me3, RNAP2, H3K27ac, H3K27me3, and RNA-seq at *HPRT1* integrated on chrX (**a, b**), and at *HPRT1R* integrated on chrX (**c, d**) and on chr3 (**e, f**), in mESCs. Two biological replicates (Rep. 1, Rep. 2) are shown for two clones (denoted (1) and (2)) derived from independent integrations. The synthetic locus region is shaded, and the *HPRT1* coding sequence is indicated in **a** and **b**. ~50 kb of flanking mouse genome is included for each position, with annotated mouse genes indicated.

**Extended Data Fig. 8:**
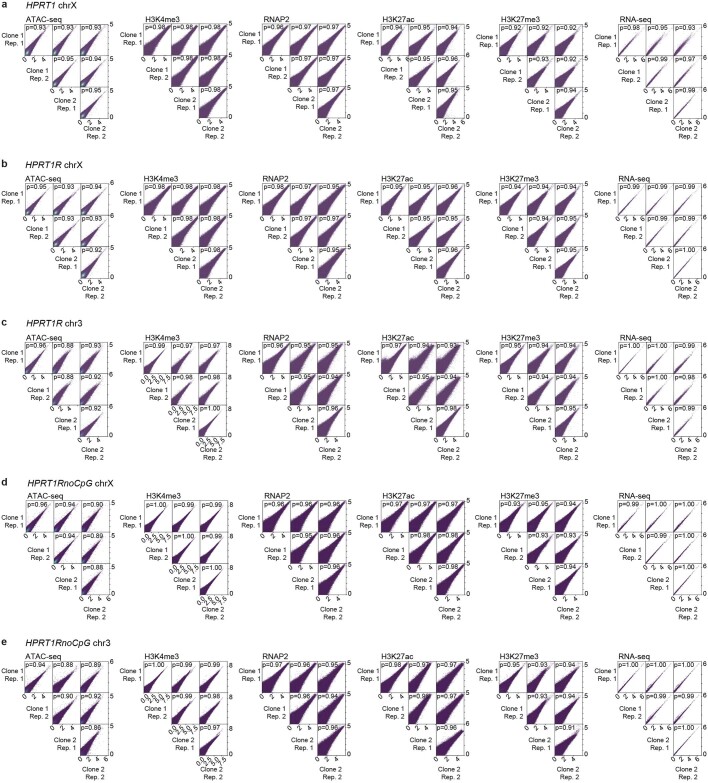
*HPRT1*, *HPRT1R* in mESCs replicate correlates. Scatterplots showing correlation between sequencing assays for *HPRT1* on chrX (**a**), *HPRT1R* on chrX (**b**), *HPRT1R* on chr3 (**c**), *HPRT1RnoCpG* on chrX (**d**), and *HPRT1RnoCpG* on chr3 (**e**). Plots compare signal, as bigWig scores, over 10 kb windows for the combined synthetic locus and mouse genome, for ATAC-seq, H3K4me3, RNAP2, H3K27ac, and H3K27me3 CUT&RUN, and RNA-seq. Both replicates (Rep. 1, Rep. 2) for each of the independent clonal isolates (Clone 1, Clone 2) are compared, and Pearson’s correlation (p) between each replicate is reported.

**Extended Data Fig. 9:**
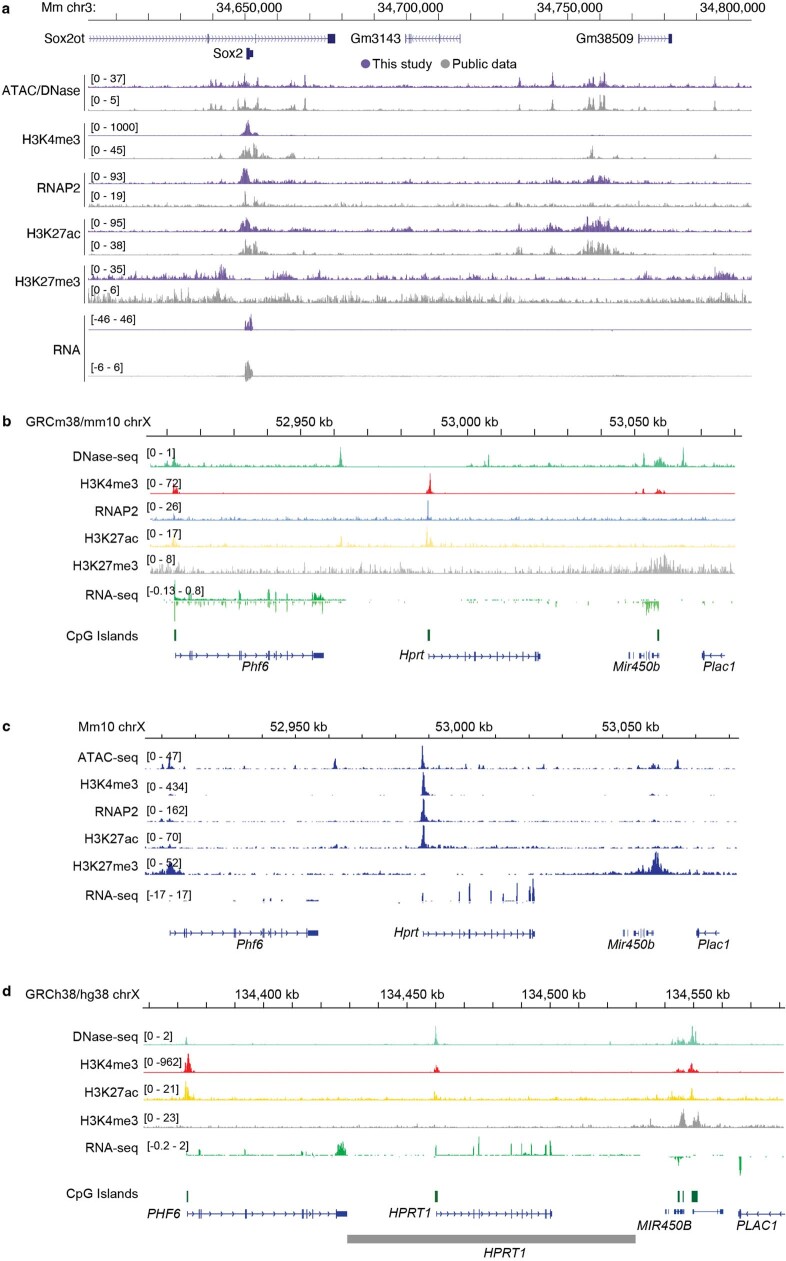
mESC sequencing assays compared to public genomic data. **a**, Snapshot of a region on *M. musculus* chromosome 3 showing sequencing data for ATAC-seq/DNase-seq, H3K4me3 ChIP-seq, H3K27ac ChIP-seq, H3K27me3 ChIP-seq, RNAP2 ChIP-seq, and RNA-seq, from this study (purple) and from publicly available, published datasets (grey). Data from this study is from one replicate mESC clone with *HPRT1* integrated on chrX. **b**, ENCODE data for DNase-seq, H3K4me3, RNAP2, H3K27ac, and H3K27me3 ChIP-seq, and RNA-seq from mouse embryonic stem cells. The browser shot shows the region on chrX used for integration of synthetic loci, including the native mouse *Hprt* gene. Predicted CpG islands are shown below. **c**, Data from this study for a clone in which *HPRT1R* in integrated on chromosome 3 (*HPRT1R* chr3 (1)) and so has an intact *Hprt* locus. **d**, ENCODE data for DNase-seq, H3K4me3, H3K27ac, and H3K27me3 ChiP-seq, and RNA-seq from the H1 human embryonic stem cell line. Browser shot is from the hg38 genome and shows the region on human chrX containing the native *HPRT1* gene. Predicted CpG islands are shown below. The region that was cloned as the synthetic *HPRT1* locus is indicated with a grey bar. Mouse datasets are: DNase-seq from ES-E14 mouse embryonic stem cells, ENCSR000CMW. ChIP-seq from ES-Bruce mouse embryonic stem cells, ENCSR000CBG, ENCSR000CDE, ENCSR000CFN, ENCSR000CCC. RNA-seq from ES-E14 mouse embryonic stem cells, ENCSR000CWC. Human datasets are: DNase-seq from H1-hESC ENCSR000EJN, ChIP-seq from H1-hESC ENCSR443YAS, ENCSR880SUY, ENCSR928HYM, RNA-seq from H1-hESC ENCSR000COU.

We next assessed the activity of synthetic loci in mouse ES cells, performing ATAC-seq, RNA-seq and CUT&RUN for RNA polymerase II (RNAP2), H3K4me3, H3K27ac and H3K27me3. Sequencing results showed high correlation between replicates (Extended Data Figs. 7 and 8), as well as similar enrichment patterns as seen in mouse ES cells previously (Extended Data Fig. 9a). For *HPRT1* integrated at *Hprt1*, we observed peaks for ATAC-seq, H3K4me3, RNAP2 and H3K27ac at the *HPRT1* TSS, and RNA-seq reads mapping specifically to the *HPRT1* exons (Fig. 3a and Extended Data Fig. 7a,b). These observations agree with public data from mouse ES cells^28^ and human ES cell lines^29^, and with data from an intact *Hprt1* locus from this study (with *HPRT1R* integrated at *Sox2*) (Extended Data Fig. 9b–d). By contrast, the *HPRT1R* locus showed no activity when integrated at this same location on the X chromosome, with no peaks for ATAC-seq, H3K4me3, H3K27ac or RNAP2 anywhere across the locus (Fig. 3b and Extended Data Fig. 7c,d). There was, however, an enrichment of H3K27me3, particularly in the flanking regions. An identical chromatin signature is observed when *HPRT1R* is integrated at *Sox2* on chromosome 3, with no peaks for ATAC-seq, H3K4me3, H3K27ac or RNAP2 and an enrichment of H3K27me3 in the flanking regions (Fig. 3c and Extended Data Fig. 7e,f). Although there is no observable transcriptional activity at *HPRT1R* in either genomic context, there is nonetheless a very small number of RNA-seq reads (0–4 across all replicates) mapping within the locus, which is less than the median of RNA-seq reads mapping to 100-kb windows of geneless regions genome-wide (10–30 mapped reads per 100-kb window) (Fig. 3d).

## *HPRT1R*^*noCpG*^ is transcriptionally silent

**Fig. 4:**
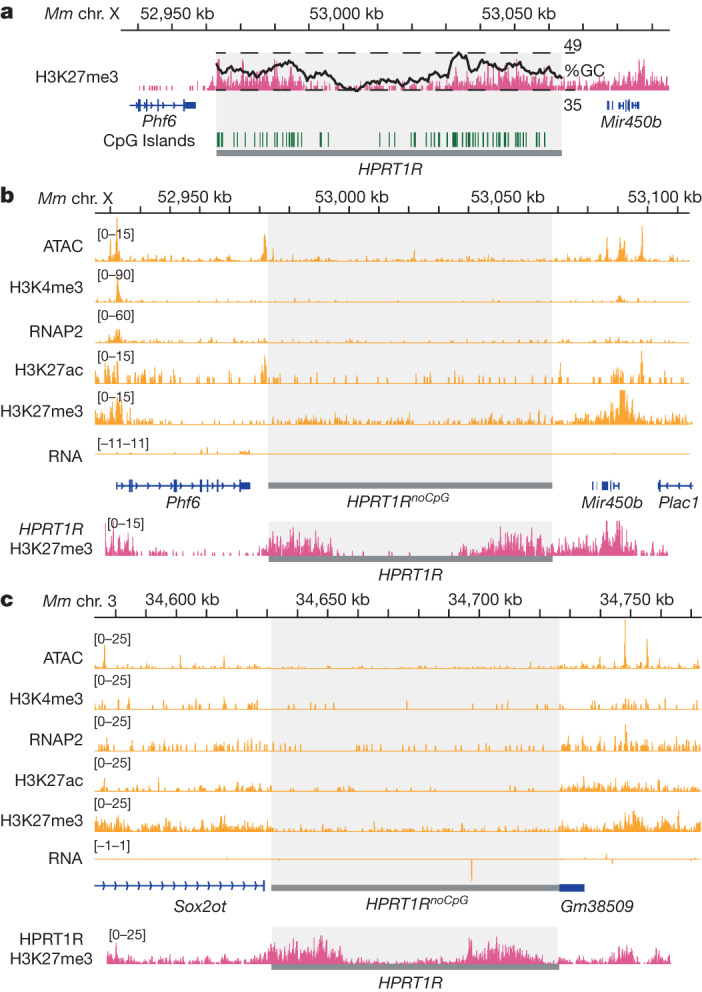
Characterization of a CpG-less HPRT1R locus. **a**, GC content overlaid with *HPRT1R* H3K27me3 CUT&RUN in mouse ES cells. GC content was calculated over 5-kb windows, and the range from 35–49% is indicated as a black line overlaying the sequencing track. CpG islands, as predicted using EMBOSS CpGplot^68^, are indicated. **b**,**c**, Sequencing tracks for ATAC-seq, H3K4me3, RNAP2, H3K27ac and H3K27me3 CUT&RUN, and RNA-seq over the *HPRT1R*^*noCpG*^ locus integrated into the mouse genome on chromosome X, clone *HPRT1R*^*noCpG*^ chromosome X (1) (**b**) and on chromosome 3, clone *HPRT1R*^*noCpG*^ chromosome 3 (1) (**c**). The synthetic locus region (*HPRT1R*^*noCpG*^) is shaded. Approximately 50 kb of flanking mouse genome is included with annotated mouse genes indicated. RNA-seq tracks are stranded, displayed with reverse strand reads inverted and below forward strand reads. The H3K27me3 CUT&RUN track for *HPRT1R* (copied from Fig. 3) is included below the respective tracks for *HPRT1R*^*noCpG*^ at each genomic position.

**Extended Data Fig. 10:**
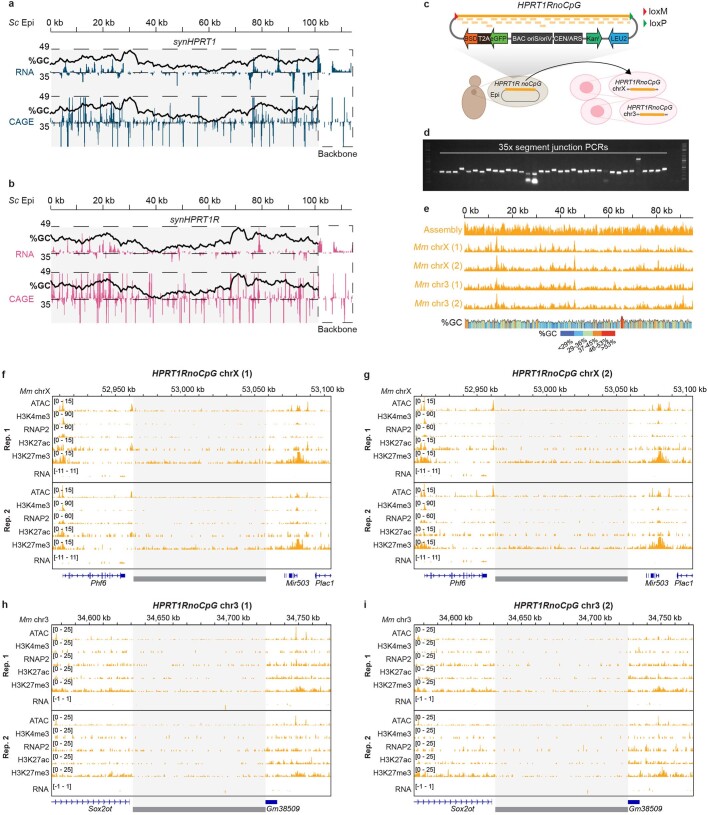
*HPRT1RnoCpG* design, assembly, and sequencing replicates. **a, b**, GC-content (%GC) overlaid with coverage tracks for episomal *HPRT1* RNA- and CAGE-seq (**a**), and episomal *HPRT1R* RNA- and CAGE-seq (**b**). %GC was calculated over 5 kb windows, and the range from 35-49% is indicated as a black line overlaying the coverage tracks. RNA-seq and CAGE-seq tracks are stranded, displayed with reverse strand reads inverted and below forward strand reads. **c**, A CpG-less version of the *HPRT1R* locus (*HPRT1RnoCpG*) was assembled from 36 segments into the same assemblon vector as *HPRT1* and *HPRT1R*. The assemblon was purified and delivered to mESCs, integrating on chrX and on chr3. **d**, PCRs across the segment junctions for the *HPRT1RnoCpG* assembly. **e**, DNA sequencing coverage plots from next generation sequencing verification of assembled and integrated synthetic loci. The yeast assembly was whole genome sequenced and mESC samples were capture-sequenced. *Assembly*, episomal assemblon in yeast; *Mm* chrX, integrated on *M. musculus* chrX; *Mm* chr3, integrated on *M. musculus* chr3 – (1) and (2) indicate two independent mESC clones; %GC, GC-content as a line plot and color-scaled. **f–i**, Sequencing tracks for ATAC-seq, H3K4me3, RNAP2, H3K27ac, H3K27me3, and RNA-seq at *HPRT1RnoCpG* integrated on chrX (**f, g**) and on chr3 (**h, i**), in mESCs. Two biological replicates (Rep. 1, Rep. 2) are shown for two clones (denoted (1) and (2)) derived from independent integrations. The synthetic locus region is shaded. ~50 kb of flanking mouse genome is included for each position, with annotated mouse genes indicated.

The synthetic *HPRT1R* locus is highly enriched relative to mammalian DNA for CpG dinucleotides (Table 1), which have been implicated in Polycomb recruitment^30–35^. This locus has increased GC content and an enrichment of CpG islands in the flanking regions compared with the middle, gene body region (Fig. 4a), corresponding to increased H3K27me3 signal in mouse ES cells and RNA-seq signal in yeast (Extended Data Fig. 10a,b). To determine whether the artificial enrichment of CpGs at this locus underlies the increased levels of H3K27me3 and low transcriptional activity, we designed a variant of *HPRT1R* in which every CpG was eliminated by randomly deleting either the C or G. This resulted in a new synthetic locus, *HPRT1R*^*noCpG*^, 5,600 bp shorter than *HPRT1R* and completely lacking CpGs. This locus was assembled de novo into the same YAV as *HPRT1* and *HPRT1R* and delivered to mouse ES cells at the *Hprt1* and *Sox2* integration sites, and the locus integrity was verified by sequencing at each step (Extended Data Fig. 10c–e). By performing the same sequencing assays as for *HPRT1* and *HPRT1R*, we found that *HPRT1R*^*noCpG*^ had lost H3K27me3 enrichment found in the flanking regions of *HPRT1R* but, notably, remained transcriptionally quiescent (Fig. 4b,c and Extended Data Fig. 10f–i).

## Discussion

By introducing synthetic reversed loci into both yeast and mouse ESCs, genomic activity across large swaths of evolutionarily naive DNA sequence can be assessed, shedding light on apparent fundamental differences in default genomic states between the two divergent cell types. In yeast, we observed widespread activity of both synthetic loci, despite the lack of promoters evolved for yeast gene expression, which did not correlate with known functional elements in the *HPRT1* locus. Previous studies in yeast have reported pervasive transcription^10,36–39^, which is generally predicted to arise from functionally specific or productive transcription. While this work was in preparation, other groups have similarly demonstrated widespread activity from an 18-kb random sequence^40^, a 244-kb sequence designed for data storage^41^, and exogenous human^42^ and bacterial^43^ chromosomes in yeast. These results, along with those from our two synthetic reversed loci, provide independent examples of active transcription in medium-to-large DNA sequences that lack evolved yeast regulatory sequences. These studies all support a hypothesis that the default state in yeast is open and active, and newly introduced loci provide ample substrates for spurious transcription initiation. Naturally, exogenous DNA may be introduced through horizontal gene transfer in the form of conventional viruses and other infectious molecules, even across relatively vast phylogenetic distances^44–47^. Yeast is largely insulated by a thick cell wall during the majority of its life cycle, and lacks conventionally transmitted viruses. Thus, yeast might be an outlier, able to afford an open, active default state to a greater extent than other eukaryotic cells.

Pervasive transcription may represent an adaptive strategy in fast-growing single-celled organisms such as yeast, in which widespread transcription of even non-coding sequences provides a chance for new, potentially favourable ‘neogenes’ to arise^48^. We observed that the RNA-seq signal was enriched in GC-rich flanking regions of both synthetic loci, possibly reflecting the increased stability of GC-rich transcripts^49–51^, and indeed new genes in yeast preferentially emerge in GC-rich intergenic regions^52^. We show here that GC-rich regions also underlie increased levels of transcription across completely non-functional loci, enabling a preview of what happens before genes are established, and generally supporting an ‘expression-first’ hypothesis^53^ for neogene formation. Following these strains harbouring synthetic loci over multiple generations may reveal whether the widespread activity seen here is tuned, or even whether novel genes can arise from such newly introduced sequences.

In contrast to yeast, activity of the novel *HPRT1R* synthetic locus was largely shut down in mouse ES cells. The *HPRT1* locus behaved as expected, faithfully recapitulating the activity of endogenous *HPRT1* in human ES cells and of *Hprt1* in mouse ES cells, indicating that the mouse ES cells have had ample opportunity to properly chromatinize the synthetic loci at the time of our analysis. *HPRT1R* showed no evidence of transcriptional activity in mouse ES cells, but did show that enrichment of H3K27me3 correlated with increased GC content and CpG islands, with an almost identical chromatin profile when integrated at two completely distinct genomic locations. It has been demonstrated previously that GC-rich sequences are important for Polycomb recruitment, particularly when the region is devoid of activating sequence motifs^30–35^. Although specific transcription factors and non-coding RNAs have also been hypothesized to have a role in Polycomb recruitment, our results are in line with observations by other groups showing that shorter sequences from *Escherichia coli*^31^ or artificial sequences designed in silico^34^, similarly devoid of mammalian TFBSs, were capable of recruiting PRC2 when integrated into the genome of mouse ES cells, suggesting that specific sequences are not required for PRC2 targeting.

Mammalian genomes are generally depleted of CpG dinucleotides^54,55^. In the *HPRT1R* locus, all GpC dinucleotides from the forward *HPRT1* are converted to CpGs, resulting in a significant 3.75-fold enrichment of this dinucleotide compared to *HPRT1*, with an observed/expected CpG ratio of 1.05 compared to 0.28 in the forward sequence. CpG dinucleotides have specifically been implicated in Polycomb recruitment^32–35^, and could explain why H3K27me3 enrichment is observed in GC-rich regions. H3K27me3 enrichment was absent in the CpG-less version of *HPRT1R*, but transcriptional activity was not restored. Thus, CpG enrichment indeed has a major role in Polycomb recruitment, but this active silencing is not responsible for the observed lack of activity. It will be informative to further modify the *HPRT1R* locus, introducing activating sequence elements such as enhancers, promoters or entire genes, and identify what elements are sufficient for introducing transcriptional activity.

The presence of extremely few RNA-seq reads mapping to the synthetic loci suggests that spurious transcription initiation is not common, and that much low-level transcription observed genome-wide may be a signal artefact or experimental noise, and does not reflect widespread functional transcription. Conversely, transcription observed at a significantly higher level may well be functional. It should be noted that these results are obtained from embryonic stem cells, and may not translate to all mammalian cells. Indeed, it has recently been reported that short random sequences inserted into *Drosophila* embryos can function as developmental enhancers, but are largely non-functional in early embryos^56^, perhaps suggesting that early embryonic genomes are generally less permissive to activity from novel sequences. As with the synthetic loci in yeast, it will be informative to measure activity arising from the *HPRT1R* locus over time, or following differentiation into different cell types that might express transcription factors that recognize chance binding sites occurring in the synthetic locus.

In comparing the same synthetic loci between the two different eukaryotic contexts, we can see substantial differences in each cell type’s requirement for transcriptional activity. Both forward and reverse loci contain thousands of TFBSs for both species, and also probably contain minor evolved sequence features, such as weak–weak base pairing and mutation periodicity around positioned nucleosomes^57^, as well as short palindromes, that would not be ablated by sequence reversal. Despite this, the loci are only broadly transcriptionally active in yeast, and we do indeed see enrichment of AT-rich stretches and short palindromes in the putative promoters, indicating that these relatively minor features may nonetheless suffice for initiating transcription. Conversely, the loci are broadly inactive in mouse ES cells, suggesting that the basic requirements for transcription in this genomic context are much more limited. Our reversed locus, although long for a sequence insertion into the mouse genome, still only represents a small fraction of an entire genome. Animal genomes are generally transcription-sparse, with even non-conserved long non-coding RNAs identified on average every 50–100 kb in the human genome^58,59^. Our reversed sequence might not be long enough for chance occurrence of a sufficiently dense cluster of proper TFBSs to initiate transcription in mouse ES cells, although the current sequence length is clearly sufficient for abundant spurious transcription in yeast cells.

The question remains of whether there are default states for gene expression. It is certainly true that the vast majority of the yeast genome is heavily transcribed and translated into proteins, whereas the opposite is true of mammalian protein-coding genes. The vast majority of animal DNA is packaged during replication into nucleosomes containing histones H3.1 and H3.2 (ref. ^60^), whereas subsequently activated regions are replaced by more yeast-like histone H3.3 nucleosomes, which are thought to be fundamentally more compatible with transcription^61^. Different genomes may respond differently to random DNA—for example, replacing yeast core nucleosomes with their human counterparts^62^ leads to a generally less permissive chromatin state, owing to intrinsically higher affinity of human nucleosomes for DNA^63,64^. However, we acknowledge that no sequence is truly random, and a future test of this hypothesis might involve evaluating thousands of sequences created by a random number generator. Indeed, as genome-engineering technologies advance, Eddy’s hypothetical multi-mega-base random genome becomes ever more plausible^13^.

In conclusion, we have used our ability to build unnatural synthetic loci as a tool to probe the default genomic states in two different eukaryotic cell types—yeast and mouse ES cells. We show that even without evolved regulatory elements, minor sequence features appear to elicit genomic activity, underlying pervasive transcription in yeast, and CpG-dependent H3K27me3 enrichment in mouse ES cells. Our results suggest that the default state in yeast is open and active, and this widespread transcription may facilitate exploitation of rare instances of horizontal transfer and provide raw fodder for neogene formation. By contrast, the default state in mouse ES cells is inactive, suggesting much more complex and limiting requirements for transcription. Here we have characterized large, evolutionarily naive sequences in different cell types, which exhibit distinct default genomic states.

## Methods

### Design of synthetic loci

The synthetic *HPRT1* locus has been described previously^18^. The synthetic *HPRT1R* locus was designed by reversing (but not reverse-complementing) the sequence of the human *HPRT1* locus corresponding to hg38 chromosome X:134429208-134529874. *HPRT1R*^*noCpG*^ was designed starting with the *HPRT1R* sequence, using a Python script to scan the sequence for occurrences of CG and randomly delete either the C or the G. As this sequence transformation can result in the formation of new CG instances, the script was reiterated until no CG sequences remained. We used software developed in house to split the synthetic loci into smaller DNA segments for commercial DNA synthesis. *HPRT1R* was split into 28 segments, 27 of ~4 kb and one of ~2 kb, and *HPRT1R*^*noCpG*^ was split into 36 segments, 35 of ~3 kb and one of 1,300 bp. Each synthetic segment had overlaps of ~300 bp, in both termini, with the neighbouring segments. MenDEL^69^ was used to design primers for junction PCR screening of yeast clones harbouring the correct assembly. Synthetic DNA segments were ordered from Qinglan Biotech, and junction PCR primers were ordered from IDT.

### Synthetic loci sequence features

Dinucleotides were counted across each synthetic locus. Expected CpG number was calculated as (no. of C × no. of G)/sequence length and CpG ratio was calculated as observed CpG/expected CpG. Yeast TFBSs were predicted by scanning the DNA sequences with the YEASTRACT+ database^65^. Mouse TFBSs were predicted using FIMO^66^ in the MEME suite using the JASPAR vertebrate motif database^67^.

### Yeast assembly and BAC recovery

All yeast work was performed starting with the parental strain BY4741 using standard yeast media. *HPRT1R* was assembled from 28 synthetic DNA segments, first as two half-assemblies that were then combined using eSwAP-In^18^. *HPRT1R*^*noCpG*^ was assembled from 36 synthetic segments in one step. For both *HPRT1R* and *HPRT1R*^*noCpG*^ assemblies, ~50 ng each of linearized and gel-purified yeast assembly vector (YAV) (pLM1110 (ref. ^17^), Addgene #168460) backbone DNA and purified assembly fragments were transformed into yeast using the high-efficiency lithium acetate method^70^. Transformants were plated on synthetic complete media lacking uracil or leucine (SC–Ura, SC–Leu) depending on the selectable marker (*URA3* for *HPRT1R* segments 1–15 half-assembly, and *LEU2* for *HPRT1R* segments 15–28 half-assembly and for *HPRT1R*^*noCpG*^ full assembly). Successful assemblies were screened by junction quantitative PCR (qPCR) on crude yeast genomic DNA (gDNA) prepared from 48 colonies from each assembly transformation. Crude yeast gDNA was prepared by performing three cycles of boiling in 20 mM NaOH at 98 °C for 3 min, followed by cooling at 4 °C for 1 min. Junction qPCRs were set up using an Echo 650 liquid handler (Labcyte) by dispensing 20 nl crude gDNA and 10 nl premixed junction primer pairs (50 µM) into a LightCycler 1536 Multiwell Plate (Roche 05358639001) containing 1 µl 1× LightCycler 1536 DNA Green mix (Roche 05573092001). qPCR reactions were performed using a LightCycler 1536 Instrument (Roche 05334276001) and successful assemblies were identified based on positive results for all junctions, defined as a having a *C*_t_ value lower than 30 (with exceptions for primer pairs determined to be consistently poor). Candidate assemblies were verified by next-generation sequencing. Libraries were prepared from 100 ng of DNA using the NEBNext Ultra II FS DNA Library Prep Kit for Illumina (NEB E7805L) with NEBNext Multiplex Oligos for Illumina (E7600S), according to the manufacturer’s protocol for FS DNA Library Prep Kit with Inputs ≤100 ng. Sequencing reactions were run on a NextSeq 500 system (Illumina SY-415-1001). Sequence-verified assemblons were recovered from yeast using the Zymoprep Yeast Miniprep I kit (Zymo Research D2001) and electroporated into TransforMax EPI300 Electrocompetent *E. coli* (Lucigen EC300150), recovered in LB + 5 mM MgCl_2_ at 30 °C for 1 h and then selected on LB + kanamycin agar plates. Bacteria colonies were screened by colony PCR for one or two assembly junctions to confirm that they contained the assemblon, then assemblon DNA was isolated from overnight cultures using ZR BAC DNA Miniprep kit (Zymo Research D4048) and verified by next-generation sequencing. eSwAP-In^18^ was used to combine the two *HPRT1R* half-assemblies. The sequence-verified assembly of segments 15–28 was purified from *E. coli* and digested with I-SceI and NotI to release the *HPRT1R* portion along with the *LEU2* marker. This digested segment was transformed into yeast harbouring the assemblon with segments 1–15, along with a Cas9–guide RNA (gRNA) expression vector, pYTK-Cas9 (ref. ^71^), with a *URA3*-targeting gRNA. The Cas9-induced break in the *URA3* marker was repaired with the *HPRT1R-*15–28-*LEU2* segment using homology provided by the common segment 15 and common sequence downstream of the selection markers. eSwAP-In transformants were selected on SC–Leu and colonies were picked to screen by junction PCR using a subset of primers spanning the entire locus. Candidate clones were verified by next-generation sequencing and recovered into *E. coli* as previously described.

The *HPRT1* locus was transplanted from its original assembly vector^18^ by restriction digestion of purified assemblon DNA with NotI and NruI to release the *HPRT1* locus, followed by co-transformation of the digested locus (~1.5 μg) along with the new, linearized, pLM1110 assembly vector (~100 ng) and linker DNAs that included *loxP* and *loxM* sites flanked by 200 bp of homology to the assembly vector and *HPRT1* locus (~50 ng each). Forty-eight colonies were picked following transformation and selection and crude yeast gDNA was screened by PCR using primers spanning the vector-*HPRT1* junctions. Candidate clones were verified by next-generation sequencing and recovered into *E. coli* as described above.

Assemblons were recovered from TransforMax EPI300 *E. coli* for delivery to mouse ES cells. Cultures of 250 ml cultures were grown at 30 °C with shaking overnight in LB + kanamycin + 0.04% arabinose to induce copy number amplification of the assemblon BAC. DNA was purified using the NucleoBond XtraBAC kit (Takara Bio 740436.25) and stored at 4 °C for less than one week before delivery to mouse ES cells.

### Integrating loci into the yeast genome

A landing pad containing a *URA3* cassette flanked by *loxM* and *loxP* sites was installed at YKL162C-A^21^ in yeast strains harbouring either *HPRT1* or *HPRT1R* assemblons. The landing pad was co-transformed, along with linker DNAs with terminal homologies to the yeast genomic locus and to the landing pad cassette (~200 ng each), into yeast as described above. Colonies were selected on SC–Ura plates, and 4 colonies were picked from each transformation and screened by PCR using primers spanning the genome–landing pad junctions. Landing pad integration was verified by Sanger sequencing of PCR products spanning the genome–landing pad junctions. The synthetic *HPRT1* and *HPRT1R* loci were integrated by Cre-mediated recombination. A *HIS3* plasmid expressing Cre-recombinase from a galactose-inducible promoter (pSH62 (ref. ^72^), Euroscarf P30120) was introduced by yeast transformation, single colonies were picked and grown to saturation in SC–His–Leu with raffinose, subcultured 1:100 in SC–His media with galactose, and plated on SC + 5-Fluoroorotic acid (5FOA) plates after 2 days of growth. 5FOA-resistant colonies were picked, screened by PCR using primers spanning the yeast genome–*HPRT1* or *HPRT1R* junctions, and verified by next-generation whole-genome sequencing as described above. Engineered yeast strains are available upon request.

### *Sphis5* insertion and transcription factor knockouts

The *His5* gene, including 5′ and 3′ untranslated regions, was cloned by PCR using Q5 high-fidelity DNA polymerase (New England Biolabs M0494L) from *S. pombe* genomic DNA. PCR primers were designed to add 40 bp of homology on each side for the desired target location in the synthetic *HPRT1* or *HRPT1R* sequence, or in the yeast genome. *Sphis5* PCR products were purified using the DNA Clean and Concentrator 5 kit (Zymo Research D4004) and transformed into *HPRT1* or *HPRT1R* episome-harbouring yeast strains, as described above. Transformations were selected on SC–His–Leu plates and correct insertions were determined by PCR using a forward primer annealing in the in the predicted promoter regions within the *HPRT1* or *HPRT1R* locus or yeast genome, outside of the homology arm, and a reverse primer annealing inside of the *Sphis5* sequence.

Select transcription factor genes were knocked out of His^+^ yeast strains by cloning the *URA3* expression cassette from pAV116 (Addgene #63183) using primers designed to add 40-bp homology arms targeting the genomic region upstream and downstream of the transcription factor coding sequence. *URA3* PCR products were purified using the DNA Clean and Concentrator 5 kit (Zymo Research D4004) and transformed into His^+^ yeast strains as above. Transformations were selected on SC–Leu–Ura and correct knockouts were verified by PCR using two sets of primers spanning the *URA3*–genome junctions.

### Yeast spot assays

Fitness of yeast strains following *Sphis5* insertions and transcription factor knockouts was assessed by spot assay. Yeast strains were grown to saturation in selective media and diluted to OD_600_ of 1 in sterile water. Five tenfold serial dilutions were made of each strain, and 5 μl of each dilution was spotted on agar plates using a multichannel pipette. Plates were incubated at 37 °C for 2 days before imaging. 3-AT, a competitive inhibitor of the *Sphis5* gene product, was used to better identify small magnitude changes in expression.

### Mouse ES cell culture

C57BL6/6J × CAST/EiJ (BL6xCAST) Δ*Piga* mouse ES cells, which enable PIGA-based Big-IN genome rewriting, have been described previously^17^. Mouse ES cells were cultured in 80/20 medium, which consists of 80% 2i medium (1:1 mixture of Advanced DMEM/F12 (ThermoFisher 12634010) and Neurobasal-A (ThermoFisher 10888022) supplemented with 1% N2 Supplement (ThermoFisher 17502048), 2% B27 Supplement (ThermoFisher 17504044), 1% GlutaMAX (ThermoFisher 35050061), 1% penicillin-streptomycin (ThermoFisher 15140122), 0.1 mM 2-mercaptoethanol (Sigma M3148), 1,250 U ml^−1^ LIF (ESGRO ESG1107l), 3 μM CHIR99021 (R&D Systems 4423), and 1 μM PD0325901 (Sigma PZ0162)), and 20% mouse ES cell medium (KnockOut DMEM (ThermoFisher 10829018) supplemented with 15% FBS (BenchMark 100106), 0.1 mM 2-mercaptoethanol, 1% GlutaMAX, 1% MEM non-essential amino acids (ThermoFisher 11140050), 1% nucleosides (EMD Millipore ES-008-D), 1% penicillin-streptomycin, and 1,250 U ml^−1^ LIF). Mouse ES cells were maintained on plates coated with 0.1% gelatin (EMD Millipore ES-006-B) at 37 °C in a humidified incubator with 5% CO_2_. C57BL6/6J × CAST/EiJ (BL6xCAST) mouse ES cells were originally provided by D. Spector, Cold Spring Harbor Laboratory, Cold Spring Harbor, NY. The BL6xCAST cell line was authenticated in next-generation capture-sequencing experiments, confirming cells as C57BL6/6J × CAST/EiJ hybrids on the basis of species-specific single-nucleotide polymorphisms. Cell lines were verified to be mycoplasma free prior to the study. There was no indication of contamination of any kind.

### Integrating synthetic loci into mouse ES cells

Integration of synthetic loci was performed using the Big-IN method^17^. First, a landing pad, LP-PIGA2, containing a polycistronic cassette, pEF1 α-PuroR-P2A-PIGA-P2A-mScarlet-EF1αpA, for selection and counterselection and flanked by *loxM* and *loxP* sites, was modified with homology arms for targeting the landing pad to the mouse *Hprt1* locus. Specifically, ~130-bp homology arms (amplified from a mouse *Hprt1* BAC) flanked by gRNA sites for the *Hprt1*-targeting gRNAs (see below) and protospacer adjacent motifs were cloned flanking the *lox* sites using BsaI Golden Gate Assembly. LP-PIGA2 was delivered to BL6xCAST Δ*Piga* mouse ES cells, along with Cas9–gRNA-expression plasmids (pSpCas9(BB)-2A-GFP, Addgene #48138) expressing gRNAs that target sites flanking the *Hprt1* locus, by nucleofection using the Neon Transfection System (ThermoFisher) as described^17^. One million cells were used per transfection with 5 μg of the landing pad plasmid and 2.5 μg each of Cas9–gRNA-expression plasmids. Cells were selected with 1 μg ml^−1^ puromycin starting day 1 post-transfection, with 6-thioguanine (Sigma-Aldrich A4660) starting day 7 post-transfection to select for the loss of *Hprt1*, and with 1 µM ganciclovir (Sigma PHR1593) to select against the landing pad plasmid backbone that contained a HSV1-ΔTK expression cassette. Candidate clones were picked on day 10, screened by qPCR using primers spanning the mouse genome–landing pad junctions and with primers for validating the loss of the endogenous *Hprt1* gene and the absence of landing pad backbone or pSpCas9 plasmid integration. Mouse ES cell clones were further verified by next-generation baited Capture-seq^17^ that the *Hprt1* locus was deleted and the landing pad was present on target. Genomic integration of a landing pad at *Sox2* has been described^20^, replacing only the BL6 allele in the hybrid BL6xCAST cell line, leaving the CAST *Sox2* allele intact. Engineered mouse ES cell lines are available upon request.

Delivery of the synthetic locus payloads was performed as described^17^ using the Amaxa 2b nucleofector (program A-23). In brief, 5 million cells were nucleofected with 5 μg pCAG-iCre (Addgene #89573) and 5 μg of assemblon DNA. Nucleofected mouse ES cells were treated with 10 µg ml^−1^ blasticidin for 2 days starting 1 day post-transfection to transiently select for the presence of the synthetic assemblons, and then with 2 nM proaerolysin for 2 days starting day 7 post-transfection to select for loss of *PIGA* in the landing pad cassette. Cells delivered with *HPRT1* were also selected with HAT medium (ThermoFisher Scientific 21060017) starting day 7 post-transfection. Clones were picked on day 9 post-transfection, expanded, and screened first by qPCR aided by an Echo 550 liquid handler (Labcyte) as described^20^ using primers spanning the junctions between the mouse genome and *HPRT1* or *HPRT1R* synthetic loci, and verified by Capture-seq^17^. For each locus integration we established two clonal cell lines from independent integration events.

### Whole-genome sequencing and Capture-seq

Whole-genome sequencing and Capture-seq were performed as previously described^17^. Biotinylated bait DNA was generated by nick translation from purified BACs and plasmids of interest: the mouse *Hprt1*- and *Sox2*-containing BACs (RP23-412J16, RP23-274P9 respectively, BACPAC Resources Center), the synthetic *HPRT1*, *HPRT1R*, and *HPRT1R*^*noCpG*^ BACs, LP-PIGA2, pCAG-iCre and pSpCas9(BB)-2A-GFP.

Sequencing and initial data processing were performed according to as previously described^17^ with modifications. Illumina libraries were sequenced in paired-end mode on an Illumina NextSeq 500 operated at the Institute for Systems Genetics. All data were initially processed using a uniform mapping pipeline. Sequencing adapters were trimmed with Trimmomatic v0.39 (ref. ^73^). Whole-genome and Capture-seq reads were aligned using BWA v0.7.17 (ref. ^74^) to a reference genome (SacCer_April2011/sacCer3 or GRCm38/mm10), including unscaffolded contigs and alternate references, as well as independently to *HPRT1* and *HPRT1R* custom references for relevant samples. PCR duplicates were marked using samblaster v0.1.24 (ref. ^75^). Generation of per base coverage depth tracks and quantification was performed using BEDOPS v2.4.35 (ref. ^76^). Data were visualized using the University of California, Santa Cruz Genome Browser. On-target, single-copy integrations are validated using DELLY^77^ call copy number variations, and bamintersect^17^ to identify unexpectedly mapping read pairs. Using these quality control steps, DELLY will identify duplications or deletions, and bamintersect will identify duplications based on read pairs mapping either between the end and the start of the synthetic locus (if duplicated in tandem) or between the synthetic locus and an unexpected genomic location (if duplicated by off-target integration). The sequencing processing pipeline is available at https://github.com/mauranolab/mapping.

### ATAC-seq

For yeast, two independent clones for each strain were inoculated into 5 ml of SC–Leu (for assemblon strains) or YPD (for integration strains) for overnight culture at 30 °C. Saturated overnight cultures were diluted to an OD_600_ of 0.1 and cultured for 6 h at 30 °C, until OD_600_ reached ~0.6. Around 5 × 10^6^ cells were taken from each culture, pelleted at 3,000*g* for 5 min, washed twice with 500 μl spheroplasting buffer (1.4 M sorbitol, 40 mM HEPES-KOH pH 7.5, 0.5 mM MgCl_2_), resuspended in 100 μl spheroplasting buffer with 0.2 U μl^−1^ zymolyase (Zymo Research E1004), then incubated for 30 min at 30 °C on a rotator. Spheroplasts were washed twice with 500 μl spheroplasting buffer then resuspended in 50 μl 1× TD buffer with TDE (Illumina 20034197). Tagmentation was performed for 30 min at 37 °C on a rotator and DNA was purified using the DNA Clean and Concentrator 5 kit (Zymo Research D4004). PCR was performed as previously described^78^ using 11 total cycles. The libraries were sequenced with 36-bp paired-end reads on a NextSeq 500 for ~1 million reads per sample.

For mouse ES cells, two independent cultures of each cell line were grown to medium confluency in 6-well plates. Cells were harvested by washing once with PBS, dissociated into single-cell suspension with TrypLE Express (ThermoFisher 12604013) and then neutralizing with equal volume mouse ES cell medium. Cells were counted and 50,000 were taken for tagmentation. Cells were pelleted at 500*g* for 5 min at 4 °C, washed with 50 μl cold PBS, resuspended in 50 μl cold ATAC lysis buffer (10 mM Tris-HCl, pH 7.4, 10 mM NaCl, 3 mM MgCl_2_, 0.1% IGEPAL CA-630), spun down at 500*g* for 10 mins at 4 °C, resuspended in 50 μl TDE mix, and incubated at 37 °C on rotator for 30 mins. DNA was purified using the DNA Clean and Concentrator 5 kit (Zymo Research D4004). PCR was performed as previously described^78^ using 10 total cycles. The libraries were sequenced with 36-bp paired-end reads for ~50 million reads per sample.

Illumina libraries were sequenced on an Illumina NextSeq 500 operated at the Institute for Systems Genetics. Sequencing adapters were trimmed with Trimmomatic v0.39 (ref. ^73^). Reads were aligned using bowtie2 v2.2.9 (ref. ^79^) to custom references in which the synthetic locus sequences were present on separate chromosomes or inserted at their specific integration sites in the SacCer_April2011/sacCer3 or GRCm38/mm10 genomes (produced using the reform tool; https://gencore.bio.nyu.edu/reform/). Coverage tracks were produced in bigWig format using bamCoverage (deepTools v3.5.0)^80^ with bin size 10 and smooth length 100, normalized using RPGC to an effective genome size of 12,000,000 for sacCer3 and 2652783500 for mm10, and visualized using IGV v2.12.3 (ref. ^81^). Peaks were called using macs2 v2.1.0 (ref. ^82^) with the parameters: --nomodel -f BAMPE --keep-dup all -g 1.2e7 (sacCer3)/1.87e9 (mm10). Relative coverage analysis was performed as described below.

### RNA-seq

For yeast, the remaining culture that was not used for ATAC-seq was centrifuged at 3,000*g* for 5 min to pellet cells, washed once with water, pelleted again at 3,000*g* for 5 min, and cell pellets were frozen at −80 °C. Frozen pellets were resuspended in 200 μl lysis buffer (50 mM Tris-HCl pH 8, 100 mM NaCl) and lysed by disruption with an equal volume of acid washed glass beads, vortexing 10× 15 s. 300 μl lysis buffer was added and samples were mixed by inversion followed by a short centrifugation to collect all liquid in the tube. Supernatant (450 μl) was mixed with an equal volume of phenol:chloroform:isoamyl alcohol, vortexed for 1 min, and centrifuged at maximum speed for 5 min. 350 μl of the aqueous layer was then mixed with an equal volume of phenol:chloroform:isoamyl alcohol, vortexed for 1 min, and centrifuged at maximum speed for 5 min. RNA was precipitated from 300 μl of the aqueous phase by adding 30 μl of 3 M sodium acetate and 800 μl of cold 99.5% ethanol, briefly vortexing, and centrifuging at maximum speed for 10 min. The pellet was rinsed with 70% ethanol and dried at room temperature before dissolving in 100 μl of RNase-free DNase set (Qiagen 79254) and incubating at room temperature for 10 min to remove DNA. RNA was purified using the RNeasy Plus Mini kit (Qiagen 74136) and eluted in 30 μl RNase-free water. RNA-seq libraries were prepared from 1 μg total RNA using the QIAseq FastSelect -rRNA Yeast kit (Qiagen 334217) and QIAseq Stranded RNA Library kit (Qiagen 180743) according to the manufacturer’s protocol. The libraries were sequenced on a NextSeq 500 with 75 bp paired-end reads for ~45 million reads per sample.

For mouse ES cells, the remaining cells that were not used for ATAC-seq were pelleted at 500*g* for 5 min and RNA was isolated using Qiagen RNeasy Plus Mini kit, resuspending in 350 μl buffer RLT Plus + β-mercaptoethanol, with homogenization using QIAshredder columns (Qiagen 79654). RNA-seq libraries were prepared from 1 μg total RNA using QIAseq FastSelect -rRNA HMR (Qiagen 334386) and QIAseq Stranded RNA kits (Qiagen 180743) according to the manufacturer’s protocol. The libraries were sequenced with 75-bp paired-end reads for ~50 million reads per sample.

Illumina libraries were sequenced on an Illumina NextSeq 500 operated at the Institute for Systems Genetics. Sequencing adapters were trimmed with Trimmomatic v0.39 (ref. ^73^). STAR (v2.5.2a)^83^ was used to align reads, without providing a gene annotation file, to custom references in which the synthetic *HPRT1* and *HPRT1R* sequences were present on separate chromosomes or inserted at their specific integration sites in the SacCer_April2011/sacCer3 or GRCm38/mm10 genomes (produced using the reform tool; https://gencore.bio.nyu.edu/reform/). Coverage tracks were produced in bigWig format using bamCoverage (deepTools v3.5.0)^80^ with bin size 10 and smooth length 100, filtering by strand, normalizing using TMM^84^, and visualized using IGV v2.12.3 (ref. ^81^). Relative coverage analysis was performed as described below.

### CUT&RUN

For yeast, two independent colonies for each strain were inoculated into 5 ml of SC–Leu (for assemblon strains) or YPD (for integration strains) for overnight culture at 30 °C. Saturated overnight cultures were diluted to OD_600_ of 0.1 and cultured for ~6 h at 30 °C, until OD_600_ reached ~0.6. Cells were pelleted at 3,000*g* for 5 min, washed twice with water, and resuspended in spheroplasting buffer (1.4 M sorbitol, 40 mM HEPES-KOH pH 7.5, 0.5 mM MgCl_2_, 0.5 mM 2-mercaptoethanol). Spheroplasting was performed by adding 0.125 U μl^−1^ Zymolyase (Zymo Research E1004) and incubating at 37 °C for 45 min on a rotator. Nuclei were prepared as previously described^85^. Resuspended nuclei were split into aliquots of ~10^8^ nuclei each and snap frozen in liquid nitrogen.

For mouse ES cells, two independent cultures for each engineered cell line cells were harvested from tissue culture dishes using TrypLE Express (ThermoFisher 12604013), dissociated into single-cell suspension, and quenched with mouse ES cell medium. Crosslinking was performed by adding formaldehyde to a final concentration of 0.1% (v/v) and incubating at room temperature for 5 min with occasional mixing by inversion. Crosslinking was stopped by quenching with 125 mM glycine and incubating at room temperature for 5 min with occasional mixing by inversion. DMSO was added to a final concentration of 10% (v/v) and cells were frozen in aliquots of ~10^6^ cells.

Isolated yeast nuclei (~10^8^ per sample) or crosslinked mouse ES cells (~10^6^ per sample) were thawed and processed for CUT&RUN using the CUTANA ChIC/CUT&RUN kit (EpiCypher 14-1048) according to the manufacturer’s protocol. Antibodies were all used at 0.5 μg: rabbit IgG negative control (EpiCypher 13-0042), H3K4me3 (EpiCypher 13-0041), H3K27ac (EpiCypher 13-0045), H3K27me3 (Active Motif 39055, RRID: AB_2561020), RNAP2 (Santa Cruz Biotechnology sc-56767). Sequencing libraries were prepared using the NEBNext Ultra II DNA Library Prep Kit for Illumina (New England Biolabs E7645L) and sequenced with 75 bp paired-end reads for ~15 M reads for H3K4me3 and Pol II samples, and ~20 M reads for H3K27ac and H3K27me3 samples.

Illumina libraries were sequenced on an Illumina NextSeq 500 operated at the Institute for Systems Genetics. Sequencing adapters were trimmed with Trimmomatic v0.39 (ref. ^73^). Reads were aligned using bowtie2 v2.2.9 (ref. ^79^) to custom references in which the synthetic *HPRT1* and *HPRT1R* sequences were present on separate chromosomes or inserted at their specific integration sites in the SacCer_April2011/sacCer3 or GRCm38/mm10 genomes (produced using the reform tool; https://gencore.bio.nyu.edu/reform/). Coverage tracks were produced in bigWig format using bamCoverage (deepTools v3.5.0)^80^ with bin size 10 and smooth length 100, normalized using RPGC to an effective genome size of 12,000,000 for sacCer3 and 2,652,783,500 for mm10, and visualized using IGV v2.12.3 (ref. ^81^). Peaks were called using macs2 v2.1.0 (ref. ^82^) with the parameters: --nomodel -f BAMPE --keep-dup all -g 1.2e7 (sacCer3)/1.87e9 (mm10). Relative coverage analysis was performed as described below.

### CAGE-seq

RNA was isolated as described above for RNA-seq, using two replicate colonies for each yeast strain. CAGE libraries were prepared as previously described^24^,starting with 5 μg RNA, with the following modifications. SuperScript IV Reverse Transcriptase (Invitrogen 18090010) was used for the reverse transcription step. AMPure XP beads (Beckman Coulter A63881) were used for all bead cleanup steps. We also used custom-made linker and primer oligonucleotides so that linkers are universal to all samples and primers contain sample-specific barcodes. Libraries were amplified using universal forward and reverse primers with 20 cycles of PCR. Libraries were sequenced on with 75 bp paired-end reads for ~22 million reads per sample.

Illumina libraries were sequenced on an Illumina NextSeq 500 operated at the Institute for Systems Genetics. Sequencing adapters were trimmed with Trimmomatic v0.39 (ref. ^73^). The 5′ reads only were aligned using bowtie2 v2.2.9 (ref. ^79^) to custom references in which the synthetic *HPRT1* and *HPRT1R* sequences were present on separate chromosomes or inserted at their specific integration sites in the SacCer_April2011/sacCer3 or GRCm38/mm10 genomes (produced using the reform tool; https://gencore.bio.nyu.edu/reform/). Coverage tracks were produced in bigWig format using bamCoverage (deepTools v3.5.0)^80^ with bin size 1, filtering by strand, normalized using RPGC to an effective genome size of 12,000,000, and visualized using IGV v2.12.3 (ref. ^81^). Peaks were called using macs2 v2.1.0 (ref. ^82^) with the parameters: --nomodel -f BAM --keep-dup all -g 1.2e7.

### Locus copy number estimation

For copy number estimation in yeast strains, coverage depth was calculated from whole-genome sequencing data for the synthetic *HPRT1* and *HPRT1R* loci as well as the entire yeast genome (excluding chrM) using samtools v1.9 depth^86^, and the calculated depth of the synthetic loci was divided by the genome average.

### Sequencing coverage analysis

Relative coverage analysis was performed for yeast ATAC-seq, RNA-seq, and CUT&RUN experiments. Average coverage depth was calculated over the synthetic *HPRT1* and *HPRT1R* loci, 100-kb sliding windows of yeast genome using samtools v1.9 bedcov^86^, which reports the total read base count (the sum of per base read depths) per specified region, and then dividing the total read base count by the region size − 100,735 bp for the *HPRT1*/*HPRT1R* loci or 100,000 bp for the 100-kb windows. Coverage was corrected for estimated copy numbers of the *HPRT1* and *HPRT1R* episomes. The yeast genome was split into 100-kb sliding windows with 10-kb step size using bedtools v2.29.2 makewindows^87^. The average of the 100-kb windows was then calculated. The average coverage depth over the synthetic loci was then divided by the relevant genome average to determine relative coverage depth in each context (that is, *HPRT1* average coverage/average coverage of yeast 100-kb windows = relative coverage of *HPRT1* compared to the yeast genome). For peak analysis, total peaks were counted across the *HPRT1* and *HPRT1R* loci, or averaged over the yeast genome 100-kb windows.

For mouse genome RNA-seq read analysis, the mouse genome was split into 100-kb sliding windows with 10-kb step size using bedtools v2.29.2 makewindows^87^. The windows were then filtered to exclude ENCODE blacklist regions^88^, centromeres, telomeres, and annotated transcripts based on Gencode comprehensive gene annotation, release M10 (GRCm38.p4). RNA-seq reads were counted for the synthetic loci and for the 100-kb genomic windows using samtools v1.9 (ref. ^86^) view with arguments -c -F 2308 -L (reference bed file).

### Replicate correlation

Correlation between sequencing assay replicates was assessed using deepTools v3.5.0 (ref. ^80^) multiBigwigSummary to first calculate average bigWig scores for each dataset across the mouse genome in 10-kb bins, and across the yeast genome in 100-bp bins. Biological and technical replicates were compared using plotCorrelation with the following arguments: --corMethod pearson --whatToPlot scatterplot --skipZeros --removeOutliers --log1p.

### Metaplots analysis

TSSs were defined as the 5′ coordinate of the experimentally identified CAGE-seq peaks. Metaplots were produced using deepTools v3.5.0 (ref. ^80^) computeMatrix and plotProfile, with argument --plotType se. Matrices were computed for ATAC-seq and H3K4me3 CUT&RUN signals and profiles were plotted for TSSs across the *HPRT1* and *HPRT1R* loci and across the rest of the yeast genome.

### Motif analysis

Putative promoter regions in the synthetic *HPRT1* and *HPRT1R* loci were defined as 200 bp upstream and 100 bp downstream of the TSSs identified based on CAGE-seq peaks (above). Motif discovery was performed on the putative promoter regions, ATAC-seq peaks, and ATAC-seq peaks that intersect with putative promoters, identified with bedtools v2.29.2 intersect^87^. Regions of interest were combined from *HPRT1* and *HPRT1R* for motif analysis using MEME v4.102 (ref. ^25^) with a maximum motif width of 10 bp. This width was determined empirically by observing that increasing widths did not result in the predicting of any more informative motifs. Tomtom^27^ was performed to scan the identified motifs for matches to motifs in the YEASTRACT database^65^. GOmo^89^ was performed to identify gene ontology terms linked to gene promoters containing the identified motifs.

### Public sequencing data

We obtained UCSC browser data for CpG islands^90,91^, as well as the following ENCODE data^92^. DNase-seq from ES-E14 mouse embryonic stem cells, ENCSR000CMW^93^. Chromatin immunoprecipitation with sequencing (ChIP-seq) from ES-Bruce mouse embryonic stem cells, ENCSR000CBG, ENCSR000CDE, ENCSR000CFN^94^, ENCSR000CCC. RNA-seq from ES-E14 mouse embryonic stem cells, ENCSR000CWC, ENCSR000CWC. ATAC-seq data from embryonic day (E)11.5 mouse embryonic tissue, ENCSR282YTE, ENCFF936VGM^28^. ChIP-seq data from E11.5 mouse embryonic tissue, ENCSR427OZM, ENCFF952ZWD, ENCSR531RZS, ENCFF033UPR, ENCSR240OUM, ENCFF179QWF^28^. DNase-seq from H1 human ES cells ENCSR000EJN, ChIP-seq from H1 human ES cells ENCSR443YAS, ENCSR880SUY, ENCSR928HYM, RNA-seq from H1 human ES cells ENCSR000COU^95^. Long RNA-seq data from H1 human ES cells, ENCSR000COU, ENCFF563OKS, ENCFF501KFP, ENCFF407PJY, ENCFF761BKF^2^.

We obtained public sequencing data for yeast from the following datasets (Gene Expression Omnibus (GEO) accession numbers): ATAC-seq (GSM6139041), H3K4me3 ChIP-seq (GSM3193266), RNA-seq (GSM5702033) and yeast CAGE-seq (ref. ^96^).

### DNA reagents

Sequences and identifiers, where applicable, for all DNA reagents used in this study are available as supplementary material, including all oligonucleotides, synthetic DNA segments, plasmids, landing pads, homology arms and yeast strains.

### Reporting summary

Further information on research design is available in the Nature Portfolio Reporting Summary linked to this article.

## Online content

Any methods, additional references, Nature Portfolio reporting summaries, source data, extended data, supplementary information, acknowledgements, peer review information; details of author contributions and competing interests; and statements of data and code availability are available at 10.1038/s41586-024-07128-2.

### Supplementary information

Supplementary Information A full guide to supplementary Tables 1–6 (tables supplied separately).

Reporting Summary

Supplementary Tables Supplementary Tables 1–6 – see Supplementary Information document for full descriptions.

## Data Availability

Data generated in this study are available in the NCBI GEO database under accession GSE252482.
