# Supplementary material for: Synthetic reversed sequences reveal default genomic states
Source: Nature. 2024 Mar 6;628(8007):373–80. doi: 10.1038/s41586-024-07128-2 (PMC11006607; doi:10.1038/s41586-024-07128-2)
Supplement: Supplementary file 1 — A full guide to supplementary Tables 1–6 (tables supplied separately). [file 41586_2024_7128_MOESM1_ESM.pdf]

nature portfolio

https://doi.org/10.1038/s41586-024-07128-2

Supplementary information

Synthetic reversed sequences reveal default genomic states

In the format provided by the authors and unedited

The accompanying Supplementary Information Excel file (**SupplementaryInformation.xlsx**) contains tabs with the Supplementary Tables outlined below.

Supplementary Table 1

Name, sequence, and length of plasmid vectors used in this work. A short description of each vectors purpose is included, as well as reference numbers for public plasmid repositories, if available.

Supplementary Table 2

Sequences of landing pads used for genomic delivery of synthetic constructs, as well as a brief description of their components, listed 5’ -> 3’, and a short description of their purpose.

Supplementary Table 3

Number, sequence, and length of synthetic DNA segments used for *de novo* assembly of *HPRT1R* and *HPRT1RnoCpG*, as well as length of overlap with the previous segment used for homology-directed repair.

Supplementary Table 4

Names and sequences of all oligos used in this work, as well as a short description of their purpose. Names of oligos used for junction PCR include the junction they are used to screen for, as well as the direction of the oligo. For example, *hHPRT1R_1-2_FP* is used to screen the *HPRT1R* assembly for the junction between segments 1 and 2 (*1-2*), and this oligo is in the forward direction (*FP*).

Supplementary Table 5

Description of homology arms used for genomic integrations of landing pads in this work. Reference genomes and specific coordinates are provided, along with specific sequences and a short description of their purpose.

Supplementary Table 6

Description of yeast strains used in this work, including their common/collection name, alias as used in this manuscript, genotype listing genomic modifications, type of YAC/BAC present, marker on the YAC/BAC, and a short description.
