# Supplementary material for: Synthetic reversed sequences reveal default genomic states
Source: Nature. 2024 Mar 6;628(8007):373–80. doi: 10.1038/s41586-024-07128-2 (PMC11006607; doi:10.1038/s41586-024-07128-2)
Supplement: Supplementary file 2 — Reporting Summary [file 41586_2024_7128_MOESM2_ESM.pdf]

nature portfolio

Corresponding author(s): Jef D. Boeke

Last updated by author(s): Jan 15, 2024

Reporting Summary

Nature Portfolio wishes to improve the reproducibility of the work that we publish. This form provides structure for consistency and transparency in reporting. For further information on Nature Portfolio policies, see our Editorial Policies and the Editorial Policy Checklist.

Statistics

For all statistical analyses, confirm that the following items are present in the figure legend, table legend, main text, or Methods section.

n/a	Confirmed	
☐	☒	The exact sample size (n) for each experimental group/condition, given as a discrete number and unit of measurement
☐	☒	A statement on whether measurements were taken from distinct samples or whether the same sample was measured repeatedly
☐	☒	The statistical test(s) used AND whether they are one- or two-sided<br>*Only common tests should be described solely by name; describe more complex techniques in the Methods section.*
☒	☐	A description of all covariates tested
☒	☐	A description of any assumptions or corrections, such as tests of normality and adjustment for multiple comparisons
☐	☒	A full description of the statistical parameters including central tendency (e.g. means) or other basic estimates (e.g. regression coefficient) AND variation (e.g. standard deviation) or associated estimates of uncertainty (e.g. confidence intervals)
☐	☒	For null hypothesis testing, the test statistic (e.g. F, t, r) with confidence intervals, effect sizes, degrees of freedom and P value noted<br>*Give P values as exact values whenever suitable.*
☒	☐	For Bayesian analysis, information on the choice of priors and Markov chain Monte Carlo settings
☒	☐	For hierarchical and complex designs, identification of the appropriate level for tests and full reporting of outcomes
☒	☐	Estimates of effect sizes (e.g. Cohen's d, Pearson's r), indicating how they were calculated

Our web collection on statistics for biologists contains articles on many of the points above.

Software and code

Policy information about availability of computer code

Data collection	No software was used for data collection.
Data analysis	Next generation sequencing data was analyzed using publicly available tools: Trimmomatic v0.39, BWA v0.7.17, samblaster v0.1.24, BEDOPS v2.4.35, bowtie2 v2.2.9, samtools v1.9, bedtools v2.29.2, deepTools v3.5.0, macs2 v2.1.0, IGV v2.12.3, meme v4.10.2.<br>Bar charts were produced using Prism 9 for macOS v9.5.0. Statistical comparison was performed using Microsoft Excel for Mac v16.66.1.

For manuscripts utilizing custom algorithms or software that are central to the research but not yet described in published literature, software must be made available to editors and reviewers. We strongly encourage code deposition in a community repository (e.g. GitHub). See the Nature Portfolio guidelines for submitting code & software for further information.

Data

Policy information about availability of data

All manuscripts must include a data availability statement. This statement should provide the following information, where applicable:

- Accession codes, unique identifiers, or web links for publicly available datasets
- A description of any restrictions on data availability
- For clinical datasets or third party data, please ensure that the statement adheres to our policy

Data generated in this study are available in the NCBI GEO database under accession GSE252482.

Human research participants

Policy information about studies involving human research participants and Sex and Gender in Research.

Reporting on sex and gender	*Use the terms sex (biological attribute) and gender (shaped by social and cultural circumstances) carefully in order to avoid confusing both terms. Indicate if findings apply to only one sex or gender; describe whether sex and gender were considered in study design whether sex and/or gender was determined based on self-reporting or assigned and methods used. Provide in the source data disaggregated sex and gender data where this information has been collected, and consent has been obtained for sharing of individual-level data; provide overall numbers in this Reporting Summary. Please state if this information has not been collected. Report sex- and gender-based analyses where performed, justify reasons for lack of sex- and gender-based analysis.*
Population characteristics	*Describe the covariate-relevant population characteristics of the human research participants (e.g. age, genotypic information, past and current diagnosis and treatment categories). If you filled out the behavioural & social sciences study design questions and have nothing to add here, write "See above."*
Recruitment	*Describe how participants were recruited. Outline any potential self-selection bias or other biases that may be present and how these are likely to impact results.*
Ethics oversight	*Identify the organization(s) that approved the study protocol.*

Note that full information on the approval of the study protocol must also be provided in the manuscript.

Field-specific reporting

Please select the one below that is the best fit for your research. If you are not sure, read the appropriate sections before making your selection.

☒ Life sciences ☐ Behavioural & social sciences ☐ Ecological, evolutionary & environmental sciences

For a reference copy of the document with all sections, see nature.com/documents/nr-reporting-summary-flat.pdf

Life sciences study design

All studies must disclose on these points even when the disclosure is negative.

Sample size	Sample sizes of 2 biological replicates for each cell line/strain were chosen as a minimum number to validate reproducibility.
Data exclusions	No data were excluded from the analyses.
Replication	2 biological replicates were performed for each cell line/strain in each experiment, with 2 technical replicates (independent clones/cell cultures) for each. All replicates agreed with each other.
Randomization	Randomization was not relevant to the study as samples did not undergo any experimental treatment.
Blinding	Blinding was not relevant during data collection as all samples were assayed with the same experimental conditions. Blinding was not possible during data analysis as sequencing files contained sample identifiers, and data had to be mapped to custom reference sequences relevant to each sample type.

Reporting for specific materials, systems and methods

We require information from authors about some types of materials, experimental systems and methods used in many studies. Here, indicate whether each material, system or method listed is relevant to your study. If you are not sure if a list item applies to your research, read the appropriate section before selecting a response.

Materials & experimental systems

n/a	Involved in the study	
☐	☒	Antibodies
☐	☒	Eukaryotic cell lines
☒	☐	Palaeontology and archaeology
☒	☐	Animals and other organisms
☒	☐	Clinical data
☒	☐	Dual use research of concern

Methods

n/a	Involved in the study	
☐	☒	ChIP-seq
☒	☐	Flow cytometry
☒	☐	MRI-based neuroimaging

Antibodies

Antibodies used	Rabbit IgG Negative Control (EpiCypher 13-0042, Lot No: 22200005-81)<br>H3K4me3 (EpiCypher 13-0041, Lot No: 22318007-81)<br>H3K27ac (EpiCypher 13-0045, Lot No: 22040004-81)<br>H3K27me3 (Active Motif 39055, Lot No: 16021022, RRID:AB_2561020)<br>Pol II (Santa Cruz Biotechnology sc-56767, Lot No: D0521)
Validation	Per EpiCypher's website: "All antibodies are validated with gold standard application-specific approaches to ensure reliable results." Validation experiments are provided for each antibody on the product-specific webpage.<br><br>Per Active Motif's website: "Antibodies are manufactured in-house, where they undergo rigorous validation procedures to ensure their quality and performance. [Their] team of scientists have also validated these antibodies for use in the applications ... such as ... ChIP-Seq ..."<br>ChIP-seq validation experiments are provided on the antibody-specific (H3K27me3) page.<br><br>Per Santa Cruz Biotechnology's website, the Pol II antibody has been cited 81 times, including IP protocols. Western blot validation is provided on the product-specific web page.<br><br>EpiCypher's CUTANA protocol suggests 0.5 µg antibody per ChIP experiment.

Eukaryotic cell lines

Policy information about cell lines and Sex and Gender in Research

Cell line source(s)	C57BL6/6J × CAST/EiJ (BL6xCAST) mESCs were originally provided by David Spector, Cold Spring Harbor Laboratory, Cold Spring Harbor, NY
Authentication	The BL6xCAST cell line is authenticated in next generation capture-sequencing experiments, confirming cells are C57BL6/6J × CAST/EiJ hybrids based on species-specific SNPs.
Mycoplasma contamination	The cell lines were not tested for mycoplasma. There was no indication of any kind of contamination.
Commonly misidentified lines<br>(See ICLAC register)	No commonly misidentified cell lines were used.

ChIP-seq

Data deposition

☐ Confirm that both raw and final processed data have been deposited in a public database such as GEO.

☒ Confirm that you have deposited or provided access to graph files (e.g. BED files) for the called peaks.

Data access links<br>*May remain private before publication.*	https://genome.med.nyu.edu/public/boekelab/HPRT1_HPRT1R_shared_data/
Files in database submission	*Provide a list of all files available in the database submission.*
Genome browser session<br>(e.g. UCSC)	https://genome.ucsc.edu/s/bcamellato/HPRT1_HPRT1R_mm10_tracks<br>https://genome.ucsc.edu/s/bcamellato/HPRT1_HPRT1R_sacCer3_tracks<br>https://genome.ucsc.edu/s/bcamellato/HPRT1_HPRT1R_track_hub

Methodology

Replicates	2 biological replicates were performed, using two independent clones for each mouse ES cell line and two different yeast strains, one with the synthetic sequence on an episome and one integrated. 2 technical replicates were performed for each cell line/strain using independent clones/cell cultures. Replicates agreed well with each other.
Sequencing depth	Sample Total reads Mapped and paired Average quality Length (bp)<br>HPRT1_HPRT1_HPRT1_mBRC216~Hprt~1-H3K27ac-BS17241A 21665458 20697336 34.4 36 Paired<br>HPRT1_HPRT1_HPRT1_mBRC216~Hprt~1-H3K27me3-BS17249A 23853996 23210486 34 36 Paired<br>HPRT1_HPRT1_HPRT1_mBRC216~Hprt~1-H3K4me3-BS17233A 15840038 14144038 34.2 36 Paired<br>HPRT1_HPRT1_HPRT1_mBRC216~Hprt~1-IgG-BS17225A 10606214 7790314 34.4 36 Paired<br>HPRT1_HPRT1_HPRT1_mBRC216~Hprt~1-PolII-BS17257A 20133316 18883604 34.3 36 Paired<br>HPRT1_HPRT1_HPRT1_mBRC216~Hprt~2-H3K27ac-BS17242A 21702010 20684368 34.3 36 Paired<br>HPRT1_HPRT1_HPRT1_mBRC216~Hprt~2-H3K27me3-BS17250A 26053158 25334738 34.3 36 Paired<br>HPRT1_HPRT1_HPRT1_mBRC216~Hprt~2-H3K4me3-BS17234A 17378218 15439176 34.2 36 Paired<br>HPRT1_HPRT1_HPRT1_mBRC216~Hprt~2-IgG-BS17226A 11320084 9199978 34.4 36 Paired<br>HPRT1_HPRT1_HPRT1_mBRC216~Hprt~2-PolII-BS17258A 19604052 17809598 34.3 36 Paired<br>HPRT1_HPRT1_HPRT1_mBRC217~Hprt~1-H3K27ac-BS17243A 24117926 23094896 34.4 36 Paired<br>HPRT1_HPRT1_HPRT1_mBRC217~Hprt~1-H3K27me3-BS17251A 25999356 25228926 34.4 36 Paired

HPRT1_HPRT1_HPRT1_mBRC217~Hprt~1-H3K4me3-BS17235A 17874308 15913278 34.3 36 Paired
HPRT1_HPRT1_HPRT1_mBRC217~Hprt~1-IgG-BS17227A 11473490 7816712 34.4 36 Paired
HPRT1_HPRT1_HPRT1_mBRC217~Hprt~1-PolII-BS17259A 21426144 19324576 34.2 36 Paired
HPRT1_HPRT1_HPRT1_mBRC217~Hprt~2-H3K27ac-BS17899A 32831410 31011302 33.7 36 Paired
HPRT1_HPRT1_HPRT1_mBRC217~Hprt~2-H3K27me3-BS17252A 25391208 24659536 34.4 36 Paired
HPRT1_HPRT1_HPRT1_mBRC217~Hprt~2-H3K4me3-BS17236A 16105174 14167044 34.2 36 Paired
HPRT1_HPRT1_HPRT1_mBRC217~Hprt~2-IgG-BS17228A 9204230 6758042 34.4 36 Paired
HPRT1_HPRT1_HPRT1_mBRC217~Hprt~2-PolII-BS17260A 20340090 18718558 34.3 36 Paired
HPRT1R_hHPRT1R_hHPRT1R~full_mBRC218~Hprt~1-H3K27ac-BS17245A 20478188 19543126 34.1 36 Paired
HPRT1R_hHPRT1R_hHPRT1R~full_mBRC218~Hprt~1-H3K27me3-BS17253A 22053376 21466308 34.4 36 Paired
HPRT1R_hHPRT1R_hHPRT1R~full_mBRC218~Hprt~1-H3K4me3-BS17237A 16258424 14347006 34.3 36 Paired
HPRT1R_hHPRT1R_hHPRT1R~full_mBRC218~Hprt~1-IgG-BS17229A 12158780 9275884 34.4 36 Paired
HPRT1R_hHPRT1R_hHPRT1R~full_mBRC218~Hprt~1-PolII-BS17261A 20819774 18796878 34.4 36 Paired
HPRT1R_hHPRT1R_hHPRT1R~full_mBRC218~Hprt~2-H3K27ac-BS17246A 21979138 20893572 34.3 36 Paired
HPRT1R_hHPRT1R_hHPRT1R~full_mBRC218~Hprt~2-H3K27me3-BS17254A 26609660 25853490 34.3 36 Paired
HPRT1R_hHPRT1R_hHPRT1R~full_mBRC218~Hprt~2-H3K4me3-BS17238A 16651588 14514940 34.3 36 Paired
HPRT1R_hHPRT1R_hHPRT1R~full_mBRC218~Hprt~2-IgG-BS17230A 9690932 6453982 34.4 36 Paired
HPRT1R_hHPRT1R_hHPRT1R~full_mBRC218~Hprt~2-PolII-BS17262A 20495150 17752444 34.4 36 Paired
HPRT1R_hHPRT1R_hHPRT1R~full_mBRC219~Hprt~1-H3K27ac-BS17247A 23179086 22193088 34.2 36 Paired
HPRT1R_hHPRT1R_hHPRT1R~full_mBRC219~Hprt~1-H3K27me3-BS17255A 24300586 23620600 34.4 36 Paired
HPRT1R_hHPRT1R_hHPRT1R~full_mBRC219~Hprt~1-H3K4me3-BS17239A 17958976 15080938 34.3 36 Paired
HPRT1R_hHPRT1R_hHPRT1R~full_mBRC219~Hprt~1-IgG-BS17231A 12798628 8002584 34.4 36 Paired
HPRT1R_hHPRT1R_hHPRT1R~full_mBRC219~Hprt~1-PolII-BS17263A 20941678 18749922 34.3 36 Paired
HPRT1R_hHPRT1R_hHPRT1R~full_mBRC219~Hprt~2-H3K27ac-BS17248A 24343656 23132290 34.2 36 Paired
HPRT1R_hHPRT1R_hHPRT1R~full_mBRC219~Hprt~2-H3K27me3-BS17256A 26795556 25976728 34.3 36 Paired
HPRT1R_hHPRT1R_hHPRT1R~full_mBRC219~Hprt~2-H3K4me3-BS17240A 17542058 14848610 34.3 36 Paired
HPRT1R_hHPRT1R_hHPRT1R~full_mBRC219~Hprt~2-IgG-BS17232A 12572468 8156830 34.4 36 Paired
HPRT1R_hHPRT1R_hHPRT1R~full_mBRC219~Hprt~2-PolII-BS17264A 21521354 18982398 34.3 36 Paired
HPRT1R_hHPRT1R_hHPRT1R~full_mBRC348~Sox2~1-H3K27ac-BS22244A 19368656 17292514 34.4 36 Paired
HPRT1R_hHPRT1R_hHPRT1R~full_mBRC348~Sox2~1-H3K27me3-BS22257A 21113202 19900326 34.5 36 Paired
HPRT1R_hHPRT1R_hHPRT1R~full_mBRC348~Sox2~1-H3K4me3-BS22231A 15256926 8129980 34.4 36 Paired
HPRT1R_hHPRT1R_hHPRT1R~full_mBRC348~Sox2~1-IgG-BS22218A 13179608 3766346 34.5 36 Paired
HPRT1R_hHPRT1R_hHPRT1R~full_mBRC348~Sox2~1-PolII-BS22270A 15772544 11334552 34.4 36 Paired
HPRT1R_hHPRT1R_hHPRT1R~full_mBRC348~Sox2~2-H3K27ac-BS22245A 20834006 18433676 34.5 36 Paired
HPRT1R_hHPRT1R_hHPRT1R~full_mBRC348~Sox2~2-H3K27me3-BS22258A 23776090 22482118 34.5 36 Paired
HPRT1R_hHPRT1R_hHPRT1R~full_mBRC348~Sox2~2-H3K4me3-BS22232A 17102232 8562486 34.5 36 Paired
HPRT1R_hHPRT1R_hHPRT1R~full_mBRC348~Sox2~2-IgG-BS22219A 13315878 4477384 34.6 36 Paired
HPRT1R_hHPRT1R_hHPRT1R~full_mBRC348~Sox2~2-PolII-BS22271A 15222544 9620940 34.5 36 Paired
HPRT1R_hHPRT1R_hHPRT1R~full_mBRC349~Sox2~1-H3K27ac-BS22246A 29913848 26442758 34.5 36 Paired
HPRT1R_hHPRT1R_hHPRT1R~full_mBRC349~Sox2~1-H3K27me3-BS22259A 21229536 20135096 34.4 36 Paired
HPRT1R_hHPRT1R_hHPRT1R~full_mBRC349~Sox2~1-H3K4me3-BS22233A 20222468 12254082 34.5 36 Paired
HPRT1R_hHPRT1R_hHPRT1R~full_mBRC349~Sox2~1-IgG-BS22220A 11691278 4004390 34.6 36 Paired
HPRT1R_hHPRT1R_hHPRT1R~full_mBRC349~Sox2~1-PolII-BS22272A 13370126 8909256 34.5 36 Paired
HPRT1R_hHPRT1R_hHPRT1R~full_mBRC349~Sox2~2-H3K27ac-BS22247A 20434500 17367916 34.4 36 Paired
HPRT1R_hHPRT1R_hHPRT1R~full_mBRC349~Sox2~2-H3K27me3-BS22260A 29143952 27354700 34.3 36 Paired
HPRT1R_hHPRT1R_hHPRT1R~full_mBRC349~Sox2~2-H3K4me3-BS22234A 17389038 8756552 34.4 36 Paired
HPRT1R_hHPRT1R_hHPRT1R~full_mBRC349~Sox2~2-IgG-BS22221A 12510504 3742298 34.5 36 Paired
HPRT1R_hHPRT1R_hHPRT1R~full_mBRC349~Sox2~2-PolII-BS22273A 16324292 10160248 34.5 36 Paired
HPRT1R_hHPRT1R_hHPRT1RnoCpG_mBRC350~Hprt~1-H3K27ac-BS22240A 15622008 12913858 34.5 36 Paired
HPRT1R_hHPRT1R_hHPRT1RnoCpG_mBRC350~Hprt~1-H3K27me3-BS22253A 22083640 20627026 34.5 36 Paired
HPRT1R_hHPRT1R_hHPRT1RnoCpG_mBRC350~Hprt~1-H3K4me3-BS22227A 15678922 8515740 34.5 36 Paired
HPRT1R_hHPRT1R_hHPRT1RnoCpG_mBRC350~Hprt~1-IgG-BS22214A 13570056 4270610 34.6 36 Paired
HPRT1R_hHPRT1R_hHPRT1RnoCpG_mBRC350~Hprt~1-PolII-BS22266A 14952698 10139458 34.4 36 Paired
HPRT1R_hHPRT1R_hHPRT1RnoCpG_mBRC350~Hprt~2-H3K27ac-BS22241A 17282152 14859906 34.4 36 Paired
HPRT1R_hHPRT1R_hHPRT1RnoCpG_mBRC350~Hprt~2-H3K27me3-BS22254A 19419410 18127992 34.5 36 Paired
HPRT1R_hHPRT1R_hHPRT1RnoCpG_mBRC350~Hprt~2-H3K4me3-BS22228A 17258502 9447216 34.5 36 Paired
HPRT1R_hHPRT1R_hHPRT1RnoCpG_mBRC350~Hprt~2-IgG-BS22215A 13167158 4003052 34.6 36 Paired
HPRT1R_hHPRT1R_hHPRT1RnoCpG_mBRC350~Hprt~2-PolII-BS22267A 14472380 9984408 34.5 36 Paired
HPRT1R_hHPRT1R_hHPRT1RnoCpG_mBRC351~Hprt~1-H3K27ac-BS22242A 17757308 15303620 34.4 36 Paired
HPRT1R_hHPRT1R_hHPRT1RnoCpG_mBRC351~Hprt~1-H3K27me3-BS22255A 22019400 20779140 34.5 36 Paired
HPRT1R_hHPRT1R_hHPRT1RnoCpG_mBRC351~Hprt~1-H3K4me3-BS22229A 19013778 11917456 34.5 36 Paired
HPRT1R_hHPRT1R_hHPRT1RnoCpG_mBRC351~Hprt~1-IgG-BS22216A 11470128 4204470 34.5 36 Paired
HPRT1R_hHPRT1R_hHPRT1RnoCpG_mBRC351~Hprt~1-PolII-BS22268A 14779752 10566236 34.3 36 Paired
HPRT1R_hHPRT1R_hHPRT1RnoCpG_mBRC351~Hprt~2-H3K27ac-BS22243A 17076856 14723290 34.4 36 Paired
HPRT1R_hHPRT1R_hHPRT1RnoCpG_mBRC351~Hprt~2-H3K27me3-BS22256A 22751050 21500238 34.4 36 Paired
HPRT1R_hHPRT1R_hHPRT1RnoCpG_mBRC351~Hprt~2-H3K4me3-BS22230A 18041610 11128594 34.4 36 Paired
HPRT1R_hHPRT1R_hHPRT1RnoCpG_mBRC351~Hprt~2-IgG-BS22217A 13196078 4912212 34.5 36 Paired
HPRT1R_hHPRT1R_hHPRT1RnoCpG_mBRC351~Hprt~2-PolII-BS22269A 15087322 10469842 34.3 36 Paired
HPRT1R_hHPRT1R_hHPRT1RnoCpG_mBRC352~Sox2~1-H3K27ac-BS22238A 21745598 19063638 34.5 36 Paired
HPRT1R_hHPRT1R_hHPRT1RnoCpG_mBRC352~Sox2~1-H3K27me3-BS22251A 24843242 23386880 34.5 36 Paired
HPRT1R_hHPRT1R_hHPRT1RnoCpG_mBRC352~Sox2~1-H3K4me3-BS22225A 18960622 11889406 34.5 36 Paired
HPRT1R_hHPRT1R_hHPRT1RnoCpG_mBRC352~Sox2~1-IgG-BS22212A 15947780 6967176 34.6 36 Paired
HPRT1R_hHPRT1R_hHPRT1RnoCpG_mBRC352~Sox2~1-PolII-BS22264A 16287034 11279224 34.4 36 Paired
HPRT1R_hHPRT1R_hHPRT1RnoCpG_mBRC352~Sox2~2-H3K27ac-BS22239A 21974598 19648288 34.5 36 Paired
HPRT1R_hHPRT1R_hHPRT1RnoCpG_mBRC352~Sox2~2-H3K27me3-BS22252A 22003352 20759686 34.6 36 Paired
HPRT1R_hHPRT1R_hHPRT1RnoCpG_mBRC352~Sox2~2-H3K4me3-BS22226A 17094392 10100370 34.5 36 Paired

HPRT1R_hHPRT1R_hHPRT1RnoCpG_mBRC352~Sox2~2-IgG-BS22213A 15981726 5457894 34.6 36 Paired
HPRT1R_hHPRT1R_hHPRT1RnoCpG_mBRC352~Sox2~2-PolII-BS22265A 19097468 12641874 34.5 36 Paired
HPRT1R_hHPRT1R_hHPRT1RnoCpG_mBRC353~Sox2~1-H3K27ac-BS22236A 18946298 16604536 34.5 36 Paired
HPRT1R_hHPRT1R_hHPRT1RnoCpG_mBRC353~Sox2~1-H3K27me3-BS22249A 22801242 21426632 34.5 36 Paired
HPRT1R_hHPRT1R_hHPRT1RnoCpG_mBRC353~Sox2~1-H3K4me3-BS22223A 17382158 11481496 34.5 36 Paired
HPRT1R_hHPRT1R_hHPRT1RnoCpG_mBRC353~Sox2~1-IgG-BS22210A 14152056 4445236 34.6 36 Paired
HPRT1R_hHPRT1R_hHPRT1RnoCpG_mBRC353~Sox2~1-PolII-BS22262A 15820542 11884942 34.3 36 Paired
HPRT1R_hHPRT1R_hHPRT1RnoCpG_mBRC353~Sox2~2-H3K27ac-BS22237A 16830752 14761594 34.5 36 Paired
HPRT1R_hHPRT1R_hHPRT1RnoCpG_mBRC353~Sox2~2-H3K27me3-BS22250A 19684274 18760184 34.5 36 Paired
HPRT1R_hHPRT1R_hHPRT1RnoCpG_mBRC353~Sox2~2-H3K4me3-BS22224A 16897214 9808368 34.5 36 Paired
HPRT1R_hHPRT1R_hHPRT1RnoCpG_mBRC353~Sox2~2-IgG-BS22211A 13679942 3712724 34.6 36 Paired
HPRT1R_hHPRT1R_hHPRT1RnoCpG_mBRC353~Sox2~2-PolII-BS22263A 14144010 9893282 34.5 36 Paired

HPRT1_Sc_H3K4me3_YAC_rep1 12990872 11961178 34.5 36 Paired
HPRT1_Sc_IgG_YAC_rep1 12942840 3068353 34.4 36 Paired
HPRT1_Sc_H3K4me3_YAC_rep2 14770434 13641102 34.4 36 Paired
HPRT1_Sc_IgG_YAC_rep2 11836572 4216222 34.4 36 Paired
HPRT1_Sc_H3K4me3_int_rep1 11296374 10498153 34.4 36 Paired
HPRT1_Sc_IgG_int_rep1 13623714 4120648 34.4 36 Paired
HPRT1_Sc_H3K4me3_int_rep2 11073750 10139640 34.4 36 Paired
HPRT1_Sc_IgG_int_rep2 13963488 4208041 34.4 36 Paired
HPRT1R_Sc_H3K4me3_YAC_rep1 14445808 14127085 34 36 Paired
HPRT1R_Sc_IgG_YAC_rep1 14633874 3561532 34.5 36 Paired
HPRT1R_Sc_H3K4me3_YAC_rep2 12439776 11382881 34.3 36 Paired
HPRT1R_Sc_IgG_YAC_rep2 13516046 4047291 34.3 36 Paired
HPRT1R_Sc_H3K4me3_int_rep1 11285006 10654608 34.4 36 Paired
HPRT1R_Sc_IgG_int_rep1 12814276 3628868 34.5 36 Paired
HPRT1R_Sc_H3K4me3_int_rep2 12519490 11412333 34.5 36 Paired
HPRT1R_Sc_IgG_int_rep2 13205994 4467406 34.5 36 Paired

Antibodies

Rabbit IgG Negative Control (EpiCypher 13-0042, Lot No: 22200005-81)
H3K4me3 (EpiCypher 13-0041, Lot No: 22318007-81)
H3K27ac (EpiCypher 13-0045, Lot No: 22040004-81)
H3K27me3 (Active Motif 39055, Lot No: 16021022, RRID:AB_2561020)
Pol II (Santa Cruz Biotechnology sc-56767, Lot No: D0521)

Peak calling parameters

Read mapping: bowtie2
Peak calling: macs2 callpeak --nomodel -f BAMPE -t $1 -g 1.87e9 --outdir $2 -n $3 --keep-dup all

Data quality

macs2 callpeak was run with default parameters, including minimum FDR of 0.05 and minimum of 50-fold enrichment.

Software

Read mapping: bowtie2
Peak calling: macs2 callpeak --nomodel -f BAMPE -t $1 -g 1.87e9 --outdir $2 -n $3 --keep-dup all
Coverage map: bamCoverage -b $1 -o $2.bw --normalizeUsing RPGC -bs 1 --effectiveGenomeSize 2652783500
Coverage depth: samtools bedcov
